# The flexible N-terminus of BchL autoinhibits activity through interaction with its [4Fe-4S] cluster and released upon ATP binding

**DOI:** 10.1074/jbc.RA120.016278

**Published:** 2020-12-03

**Authors:** Elliot I. Corless, Syed Muhammad Saad Imran, Maxwell B. Watkins, John-Paul Bacik, Jenna R. Mattice, Angela Patterson, Karamatullah Danyal, Mark Soffe, Robert Kitelinger, Lance C. Seefeldt, Sofia Origanti, Brian Bennett, Brian Bothner, Nozomi Ando, Edwin Antony

**Affiliations:** 1 Department of Biological Sciences, Marquette University, Milwaukee, Wisconsin, USA; 2 Department of Biochemistry, Saint Louis University School of Medicine, St Louis, Missouri, USA; 3 Department of Chemistry, Princeton University, Princeton, New Jersey, USA; 4 Department of Chemistry and Chemical Biology, Cornell University, Ithaca, New York, USA; 5 Department of Chemistry and Biochemistry, Montana State University, Bozeman, Montana, USA; 6 Department of Chemistry and Biochemistry, Utah State University, Logan, Utah, USA; 7 Department of Biology, Saint Louis University, St Louis, Missouri, USA; 8 Department of Physics, Marquette University, Milwaukee, Wisconsin, USA

**Keywords:** electron transfer, nitrogenase, nitrogenase-like enzymes, DPOR, photosynthesis, iron–sulfur cluster, Chlide, chlorophyllide, COR, chlorophyllide oxidoreductase, DPOR, dark-operative protochlorophyllide oxidoreductase, ET, electron transfer, FA, formic acid, HDX-MS, Hydrogen deuterium exchange mass spectrometry, Pchlide, protochlorophyllide

## Abstract

A key step in bacteriochlorophyll biosynthesis is the reduction of protochlorophyllide to chlorophyllide, catalyzed by dark-operative protochlorophyllide oxidoreductase. Dark-operative protochlorophyllide oxidoreductase contains two [4Fe-4S]–containing component proteins (BchL and BchNB) that assemble upon ATP binding to BchL to coordinate electron transfer and protochlorophyllide reduction. But the precise nature of the ATP-induced conformational changes is poorly understood. We present a crystal structure of BchL in the nucleotide-free form where a conserved, flexible region in the N-terminus masks the [4Fe-4S] cluster at the docking interface between BchL and BchNB. Amino acid substitutions in this region produce a hyperactive enzyme complex, suggesting a role for the N-terminus in autoinhibition. Hydrogen–deuterium exchange mass spectrometry shows that ATP binding to BchL produces specific conformational changes leading to release of the flexible N-terminus from the docking interface. The release also promotes changes within the local environment surrounding the [4Fe-4S] cluster and promotes BchL-complex formation with BchNB. A key patch of amino acids, Asp-Phe-Asp (the ‘DFD patch’), situated at the mouth of the BchL ATP-binding pocket promotes intersubunit cross stabilization of the two subunits. A linked BchL dimer with one defective ATP-binding site does not support protochlorophyllide reduction, illustrating nucleotide binding to both subunits as a prerequisite for the intersubunit cross stabilization. The masking of the [4Fe-4S] cluster by the flexible N-terminal region and the associated inhibition of the activity is a novel mechanism of regulation in metalloproteins. Such mechanisms are possibly an adaptation to the anaerobic nature of eubacterial cells with poor tolerance for oxygen.

Photosynthetic organisms utilize chlorophyll or bacteriochlorophyll to capture light for their energy requirements. The multistep enzymatic biosynthesis of both these compounds relies on similar pathways in the cell. One key exception is the enzyme utilized for the reduction of protochlorophyllide (Pchlide) to chlorophyllide (Chlide), a key intermediate in the biosynthetic pathway (1, 2). Angiosperms use a light-dependent Pchlide oxidoreductase to catalyze the reduction, whereas gymnosperms, cyanobacteria, algae, bryophytes, and pteridophytes possess a light-independent enzyme called dark-operative protochlorophyllide oxidoreductase (DPOR; Fig. 1*A*) (3). Photosynthetic bacteria that are anoxygenic rely exclusively on the activity of DPOR for synthesis of bacteriochlorophyll (3). DPOR catalyzes the stereospecific reduction of the C17 = C18 double bond of Pchlide, a porphyrin, to form Chlide, a chlorin (Fig. 1*B*), leading to a parental shift in the spectral properties required for photosynthesis (4, 5).

**Figure 1:**
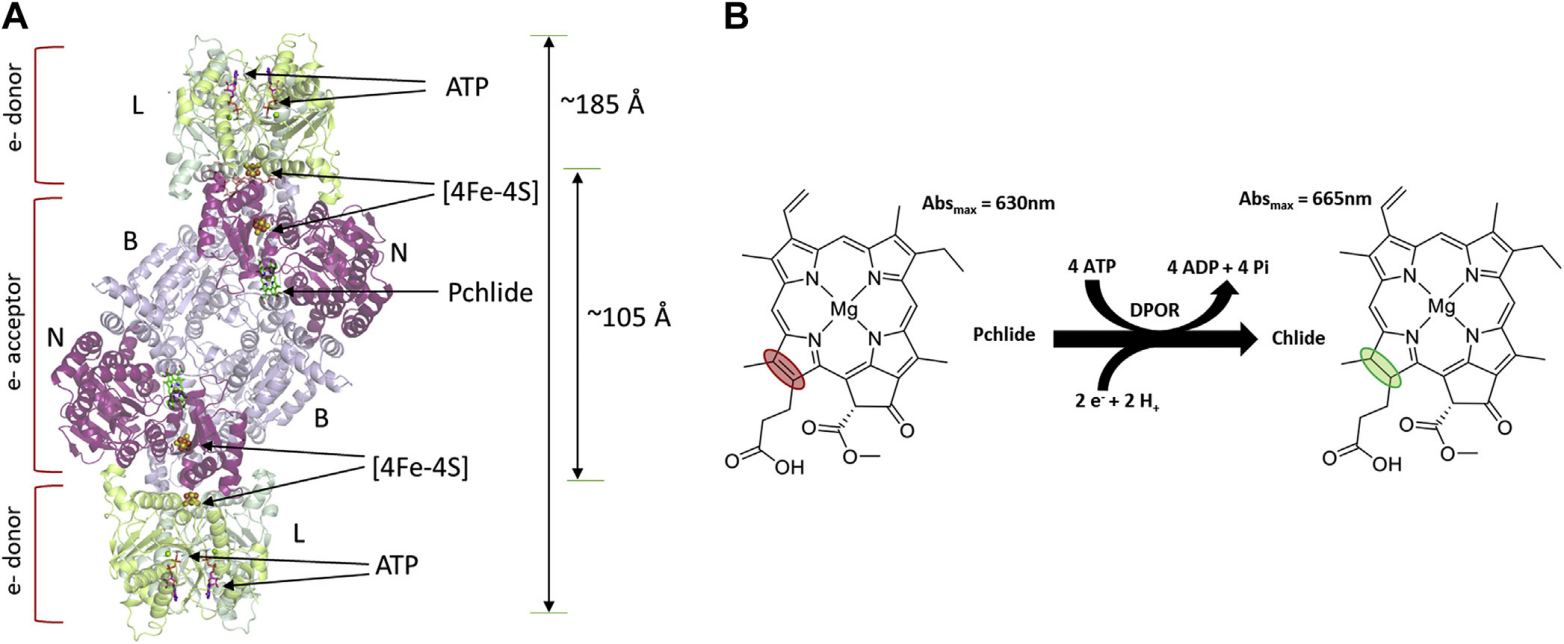
**Structure and substrate reduction mechanism of DPOR.***A*, crystal structure of the complete, ADP–AlF_3_–stabilized, DPOR complex from *Prochlorococcus marinus* (PDB ID: 2YNM). BchL subunits are colored *green*, BchN is colored *purple*, and BchB is colored *violet*. The four [4Fe-4S] clusters are shown as *spheres*, and ADP–AlF_3_ and Pchlide are shown as *sticks*. *B*, the scheme of Pchlide reduction to Chlide by DPOR. Two cycles of electron transfer from BchL to BchNB are required for the reduction of the C17 = C18 double bond (marked by the colored *ovals*). DPOR, dark-operative protochlorophyllide oxidoreductase; Chlide, chlorophyllide.

Interestingly, DPOR is structurally homologous to nitrogenase, the enzyme responsible for reducing dinitrogen to ammonia (6, 7). Like nitrogenase, DPOR is composed of two proteins: a homodimeric L-protein (BchL) and a heterotetrameric NB-protein (BchNB) (5, 8). BchL serves as the ATP-dependent electron donor, and BchNB is the electron acceptor containing the active site for Pchlide binding and reduction (Fig. 1*A*). Reducing equivalents of electrons are donated from ferredoxin to BchL. A multistep reaction cycle has been proposed for DPOR with the following overall reaction stoichiometry (Fig. 1*B*) (4):

$$\text{Pchlide }+\;4\text{ATP }+\;2{\text{e}}^{-}\;+\;2{\text{H}}^{+}\;+\;4{\text{H}}_{2}\text{O }\to \text{ Chlide }+\;4\text{ADP }+\;4{\text{P}}_{\text{i}}$$

Two such rounds of ATP-dependent electron transfer (ET) are necessary for Pchlide reduction, and the minimum stoichiometry of ATP/molecule of Chlide formed has been determined to be 4 (9). Given the structural similarity to nitrogenase, ATP binding to BchL is thought to promote its transient association with BchNB followed by a single ET to Pchlide. ATP hydrolysis drives the dissociation of the protein complex (9, 10, 11, 12). However, the details of how ATP binding promotes complex formation between BchL and BchNB are poorly resolved.

Unlike nitrogenase, however, only two high-resolution crystal structures of the L-protein dimer have been reported: one in which the BchL dimer is complexed with ADP (PDB ID: 3FWY *Rhodobacter sphaeroides* BchL) (10) and another in which it is stabilized in a higher-order complex with the BchNB tetramer by the transition-state analogue, ADP–AlF_3_ (PDB ID: 2YNM *Prochlorococcus marinus DPOR*; Fig. 1*A*) (11). These structures reveal that BchL contains one [4Fe-4S] cluster at the dimer interface ligated by 2 conserved cysteine residues from each subunit (Fig. 1*A*). Each monomer of BchL contains an active site for ATP, including conserved phosphate-binding (Walker A, residues 38–45 in *R. sphaeroides* BchL) and hydrolysis (Walker B, residues 154–158) motifs. Each half of the BchNB tetramer contains a substrate (Pchlide) binding site and one [4Fe-4S] cluster that functions as the electron acceptor from BchL (Fig. 1*A*). This cluster is ligated by 3 cysteine residues from BchN and one uncommon aspartic acid ligand from BchB. In the structure stabilized with ADP–AlF_3_, the L-protein sits across the top of BchNB, placing their metal clusters in relative proximity (∼16 Å; Fig. 1*A*) (11). Thus, ATP binding is hypothesized to drive formation of the complex and ET, whereas ATP hydrolysis has been proposed to drive dissociation of the BchL–BchNB complex (11, 13). Additional functional control of DPOR activity is imparted through asymmetric Pchlide binding and ET events within the tetramer (14).

In the homologous nitrogenase system, ATP hydrolysis occurs after the ET, suggesting that hydrolysis likely drives complex dissociation after the ET (12, 15). Given the structural similarities between DPOR and nitrogenase, we hypothesize that ATP hydrolysis similarly promotes complex dissociation in DPOR.

Here, we address three outstanding questions about ATP usage by BchL: How does binding of 2 ATP molecules collectively transmit information from the ATP binding sites to the [4Fe-4S] cluster of BchL along with the interface where it complexes with BchNB? What role does ATP play in ET? Are both ATP-binding events necessary?

We present the first crystal structure of *R. sphaeroides* BchL in the nucleotide-free state. This structure reveals a novel electron density for a flexible N-terminal region that is bound across the face of the BchL [4Fe-4S] cluster, suggesting a potential regulatory role. We show that amino acid substitutions within this flexible N-terminal region enhance the kinetics of Pchlide reduction, pointing to a functionally autoinhibitory role. We find that intersubunit contacts between BchL and bound ATP are critical for Pchlide reduction activity and that ATP binding promotes release of the inhibition through allosterically coordinated conformational changes. Together, our results support a model where ATP-driven cross stabilization of the homodimer promotes the release of the flexible N-terminus and allows formation of the BchL–NB complex activating ET and Pchlide reduction.

## Results

### Crystal structure of nucleotide-free BchL suggests regulation through a flexible N-terminal region

**Figure 2:**
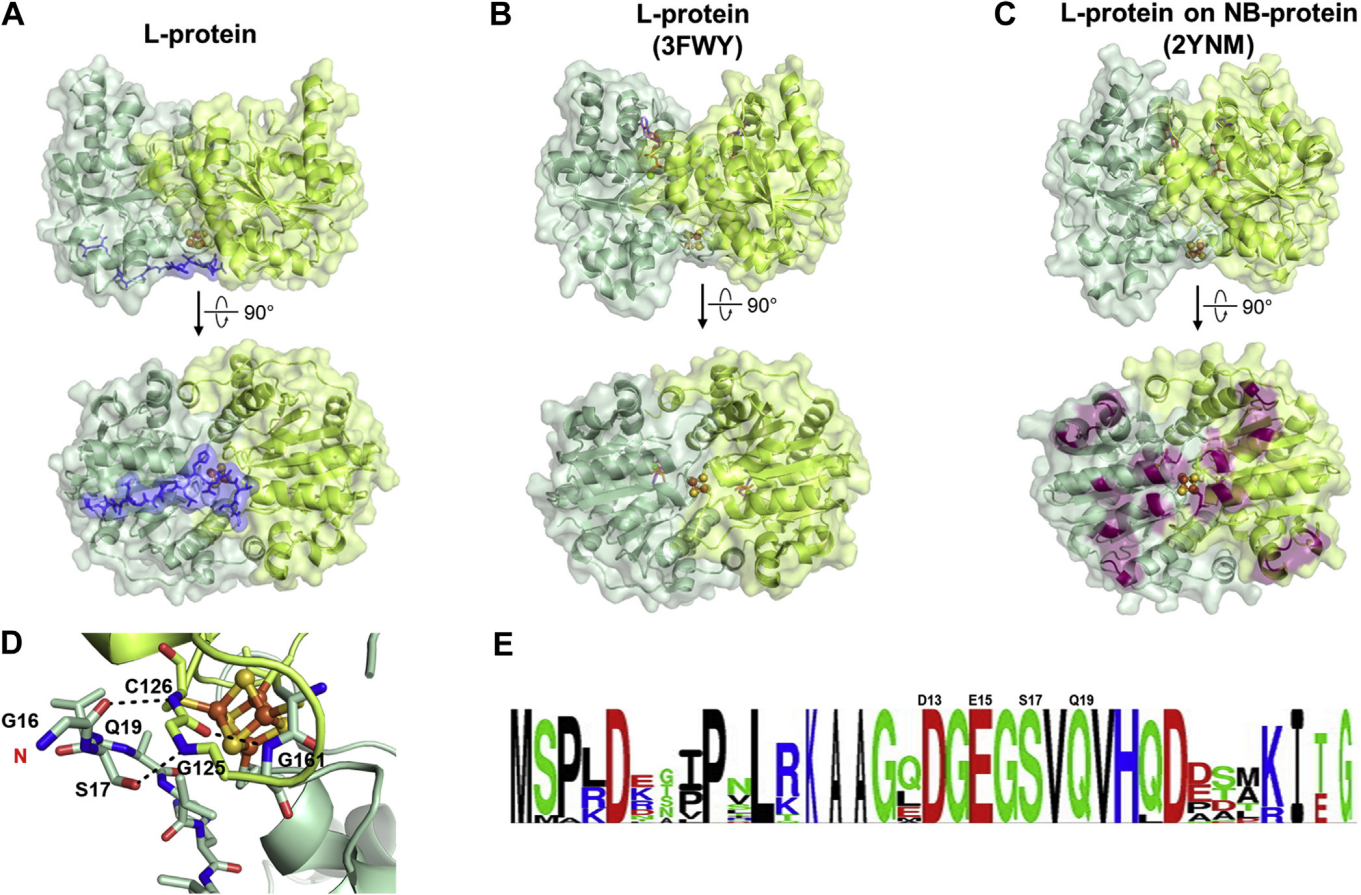
**Crystal structure of nucleotide-free BchL reveals a flexible N-terminal region capping the [4Fe-4S] cluster.** Side (*top row*) and bottom (*bottom row*) views of the BchL structure (*A*) in the absence of nucleotides, (*B*) with ADP bound (*R. sphaeroides*, PDB ID: 3FWY), and (*C*) in the complex with ADP–AlF_3_ and BchNB (*Prochlorococcus marinus*, PDB ID: 2YNM; NB-protein not shown). A slight compaction upon the addition of ADP and a further, more significant compaction when in complex with ADP–AlF_3_–BchNB can clearly be seen by comparing the side views. The flexible N-terminal region resolved in the nucleotide-free structure (residues 16–29, colored *blue* in panel *A*) clearly covers the [4Fe-4S] cluster in addition to blocking or directly interacting with several residues predicted to directly interact with BchNB (highlighted in *dark purple* in panel *C*). *D*, residues in the flexible N-terminus of chain C interact both directly and indirectly with important residues near the [4Fe-4S] cluster. *E*, sequence logo of the N-terminus of L-protein generated from alignment of n = 89 species. The letter height corresponds to the degree of sequence conservation, and the residues mutated in this study are noted.

*R. sphaeroides* BchL was crystallized anaerobically in the absence of nucleotides, and a crystal structure was determined to a resolution of 2.6 Å (Table S1). The asymmetric unit contains four BchL chains: chains A and B form one BchL dimer and chains C and D comprise the other (Fig. S1*A*). The overall conformations of the AB and CD dimers are highly similar (Cα RMSD, of 0.55 Å). Comparison of the nucleotide-free CD dimer with previous BchL structures (Fig. 2, *A*–*C*) reveals an increasing compaction in the top face of the dimer from the nucleotide-free state to ADP-bound BchL (PDB ID: 3FWY, Cα RMSD = 1.62 Å) to ADP–AlF_3_–NB–bound L-protein from *P. marinus* (11) (PDB ID: 2YNM, Cα RMSD = 3.98 Å).

Unexpectedly, although the N-terminus is disordered in previous DPOR L-protein structures (residues 1–29 in 3FWY and residues 1–27 in 2YNM), we observe the electron density at the N-terminus of chain C in our nucleotide-free structure, which we were able to model as residues 16 to 29 (Fig. 1*B*). Although the electron density for this region is not observed in the other chains, superimposing chain C onto chain D reveals that only one N-terminus would be able to bind in the observed conformation because of steric clashes (Fig. S1*C*). The lack of observable density in this region for the A chain may be due to the close proximity to a crystallographically related chain D, whereas the flexible nature of the N-terminus may nonetheless preclude its observation in all but the C chain.

Interestingly, in chain C, we observe the flexible N-terminus bound across the [4Fe-4S] cluster (Fig. 2*A*, *dark blue*) covering a surface that is normally used to interface with BchNB (Fig. 2*C*, *purple*). This conformation appears to be stabilized through both intrasubunit and intersubunit interactions in the homodimer. For example, in chain C, Asp-23 forms a hydrogen bond with Gln-168 (bond distance of 2.8 Å), whereas the backbone oxygen of Gly-16 (chain C) is within hydrogen-bonding distance of the Cys-126 (chain D) backbone amide (3.5 Å), a [4Fe-4S] cluster-ligating residue. In addition, the side chain of Ser-17 (chain C) is well positioned to interact with the residue directly adjacent to Cys-126, Gly-125 of chain D, through a hydrogen bond with the backbone amide (3.5 Å, Fig. 2*D*, Fig. S1*B*). The backbone oxygen of Gly-125 in chain D in turn is within hydrogen-bonding distance of the Gly-161 backbone (3.0 Å) in chain C. Based on the structure of the ADP–AlF_3_–stabilized DPOR complex (PDB ID: 2NYM) (11), the residues corresponding to Cys-126, Gly-161, and Gln-168 in BchL (*R. sphaeroides* numbering) are three of the twelve residues (per monomer) that interact with BchNB during the formation of the active complex, as assessed by PDBePISA interface analysis (16) (Fig. 2*C*, *purple* surface).

Notably, the flexible N-terminal region (residues 1–29) is only conserved among DPOR BchL proteins and is not observed in other homologous proteins such as the nitrogenase Fe protein and the BchX protein of chlorophyllide oxidoreductase (COR) (Fig. S2). The position and specific interactions of the N-terminal residues in our nucleotide-free structure suggest a possible autoinhibitory role by forming a barrier to docking and shielding the [4Fe-4S] cluster of BchL.

### Amino acid substitutions in the flexible N-terminal region of BchL increases the rate of Pchlide reduction

To test whether the flexible N-terminal region of BchL plays an autoinhibitory role, based on the contacts observed in the crystal structure (Fig. 2*D*) and sequence alignments (Fig. 2*E*), we generated a singly mutated construct (BchL^S17A^) where Ser-17 was substituted to Ala, and a quadruply mutated construct (BchL^4A^) where Asp-13, Glu-15, Ser-17, and Gln-19 were all substituted with Ala. Although density for residues 1 to 15 is not seen in any BchL structure, Ser-17 and Gln-19 are positioned across the [4Fe-4S] cluster in our structure (Fig. S1*B*, the side chain of Gln-19 is disordered).

**Figure 3:**
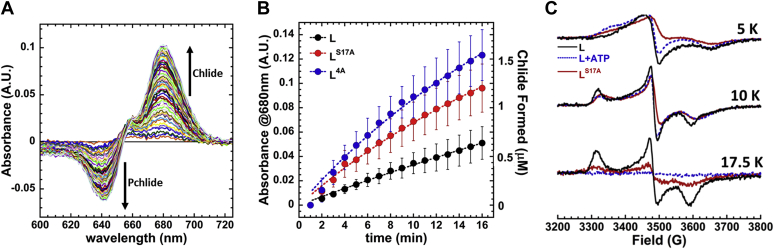
**The flexible N-terminal region is autoinhibitory to BchL function.***A*, representative traces of a WT DPOR reduction reaction in the aqueous solution. Traces represent subtracted data using the zero time point as reference. Thus, Pchlide (substrate) and Chlide (product) absorbance signals trend in the opposite directions denoted by *up* and *down arrows*, respectively. *B*, the plot of the *in vitro* reduction rates as a change in absorbance at 680 nm, A_680_, (*left axis*) and the amount of Chlide formed (*right axis*). Data were fit to a single exponential and yield the following observed rates: BchL (*black circles*) *k*_*obs*_= 0.026 ± 0.002 min^-1^), BchL^S17A^ (*red circles*) *k*_*obs*_ = 0.041 ± 0.008 min^-1^, and BchL^4A^ (*blue circles*) *k*_*obs*_ = 0.045 ± 0.007 min^-1^. *C*, EPR spectra comparing BchL (*black solid lines*), BchL incubated with excess ATP (*blue dotted lines*), and BchL^S17A^ (*red solid lines*) at 5 K, 10 K, and 17.5 K as indicated. DPOR, dark-operative protochlorophyllide oxidoreductase; Chlide, chlorophyllide; Pchlide, protochlorophyllide.

Pchlide-reducing activity with purified protein was assayed by mixing purified BchL, BchL^S17A^, or BchL^4A^ (4 μM) with BchNB (1 μM) and Pchlide (35 μM) and spectroscopically monitoring Chlide formation over time in the absence or presence of ATP (3 mM). Pchlide and Chlide have characteristic absorbance maxima at 630 nm and 680 nm, respectively, in the aqueous solution. Formation of Chlide was observed as an increase in absorbance at 680 nm in the presence of ATP (Fig. 3*A*). Both BchL^S17A^ and BchL^4A^ are active for Pchlide reduction and show Chlide formation rates ∼2-fold higher than that of WT BchL (k_obs_= 0.026 ± 0.002, 0.041 ± 0.008, and 0.045 ± 0.007 min^-1^ for BchL, BchL^S17A^, and BchL^4A^, respectively; Fig. 3*B*).

We also generated a truncated version of BchL missing the first 27 amino acids (BchL^NΔ27^). However, it was poorly soluble, and we were unable to obtain sufficiently pure protein for biochemical studies. *In vitro*, in Pchlide reduction reactions, when ATP is added, we notice a small degree of precipitation during the course of the experiment (∼30 min). Thus, we speculate that actual differences in the activity are likely much larger as the effective concentrations of the variant BchL proteins are lower than those calculated at the start of the experiment because of protein instability. These observations provide additional evidence that the flexible N-terminus may play an important role in BchL function and stability.

## ATP-binding causes changes in the local environment of the BchL [4Fe-4S] cluster affecting its EPR spectral line shape and intensities

Since the flexible N-terminal region binds to the BchNB interaction interface within BchL, we hypothesized that ATP binding could promote conformational changes in BchL to relieve autoinhibition. We thus used electron paramagnetic resonance (EPR) spectroscopy to probe local changes in the [4Fe-4S] cluster environment, which includes the binding site for the N-terminus. Interpretation of the g-tensors of [4Fe-4S] clusters in direct structural terms is rarely possible (17), but changes in the EPR line shape can result from electronic changes in the cluster, which can be caused by conformational changes around the cluster. Changes in intensity reflect changes in the oxidation state. Previous studies have described the [4Fe-4S] cluster of BchL as an axial species (5, 8, 18), whereas others reported a rhombic species (13, 19). Here, EPR at different temperatures indicated that two distinct EPR signals were exhibited by the [4Fe-4S] cluster (Fig. S3*A*). At 5 K, a signal termed FeS^A^ was observed that appears axial but was best simulated with rhombic *g*-values of 1.98, 1.94, and 1.85; both *g*_1_ and *g*_3_ are atypically low for a prototypical Cys_4_-ligated [4Fe-4S] cluster, and the associated resonances exhibit large line widths (Fig. S3*A*). This signal was very fast-relaxing and was no longer detectable at 17.5 K. At this higher temperature (17.5 K), a more typical rhombic signal, FeS^B^, with *g*_1,2,3_ = 2.04, 1.94, and 1.89 was observed, and at an intermediate temperature (10 K), the observed signal was well replicated by a 40%:60% mixture of the simulations of FeS^A^ and FeS^B^, respectively. As expected for a [4Fe-4S] cluster, the EPR signals were fast-relaxing and were not detectable at 30 K or a higher temperature.

BchL exhibited analogous signals to FeS^A^ and FeS^B^ when incubated with BchNB (Fig. S3*B*) and ADP (Fig. S3*C*), although, notably, both ADP and BchNB served to increase the relaxation rate of the FeS^B^ EPR signal, suggesting a more efficient coupling of the cluster to the lattice *via* increased strain energy. In addition, with ADP, the proportion of the FeS^A^ species was diminished by a factor of two. Based on the structural data for BchL and the relaxation properties of the EPR signals, we propose the FeS^A^ species as having a 'cap' across the cluster, formed by the flexible N-terminus, whereas the FeS^B^ species is uncapped; in solution, these two species are likely in dynamic equilibrium. Upon the addition of ATP, the relaxation rate of the FeS^B^ species was further enhanced and was undetectable at 17.5 K (Fig. S3*C*), whereas the FeS^A^ signal exhibited rapid-passage distortion at 5 K, indicating a diminution of the relaxation rate for that species. These data suggest that ATP binding increases the conformational strain of uncapped FeS^B^ and somewhat inhibits the strong interaction of the cap with the cluster in FeS^A^. The FeS^A^ EPR signals from the BchL^S17A^ variant (Fig. S3*D*) exhibited strong rapid-passage distortion at 5 K and overall reduced signal intensities over the 5- to 17.5-K temperature range; the addition of ATP restored the intensity of the FeS^B^ signal somewhat, suggesting that relaxation properties were responsible for this phenomenon and that, therefore, the interaction of the cap region with the cluster (and/or associated conformational changes) is altered in BchL^S17A^. The EPR signal at 17.5 K highlights the major ATP-driven conformational differences between BchL and BchL^S17A^. For BchL, the two peaks observed in the absence of ATP are significantly reduced upon ATP binding (compare *black* and *red* traces in Fig. S3*C*). For BchL^S17A^, the peaks are not present in the absence or presence of ATP (compare *red* and *blue* traces in Fig. S3*D*). Thus, it is likely that the conformational changes around the [4Fe-4S] cluster in BchL upon ATP binding are similar to those of the BchL^S17A^ protein in the absence of ATP (Fig. 3*C*). The mutagenesis and EPR data lead us to propose that the loss of autoinhibition drives higher overall Pchlide reduction activity in BchL^S17A^. To test this model, hydrogen–deuterium exchange mass spectroscopy (HDX-MS) experiments were performed to capture the ATP binding–induced conformational changes in BchL and BchL^S17A^.

## HDX-MS reveals ATP binding–driven conformational changes in BchL that promote release of the N-terminal flexible region from the BchNB docking region

The EPR data suggest ATP-driven conformational changes in the protein, especially around the [4Fe-4S] cluster region and an ATP-binding driven mechanism for its release from the [4Fe-4S] cluster and the BchNB binding interface. To obtain a direct readout of such global conformational changes in BchL, we used HDX-MS to compare ATP binding–driven changes in BchL and BchL^S17A^. HDX-MS measures the rate of amide-H exchange to report on changes in the local solvent environment (20). D_2_O exchange rates are shaped by dynamics, H-bonding, secondary structure, and solvent exposure. Increased rates of D_2_O exchange result from greater solvent accessibility, disruption in backbone H-bonding, or increased dynamics (21). Decreased D_2_O exchange rates thus suggest less solvent accessibility or local stabilization (*i.e.,* H-bonding and/or formation of secondary structure). As such, exchange rate disruptions are valuable indicators of protein allostery, dynamics, and interfaces (22).

**Figure 4:**
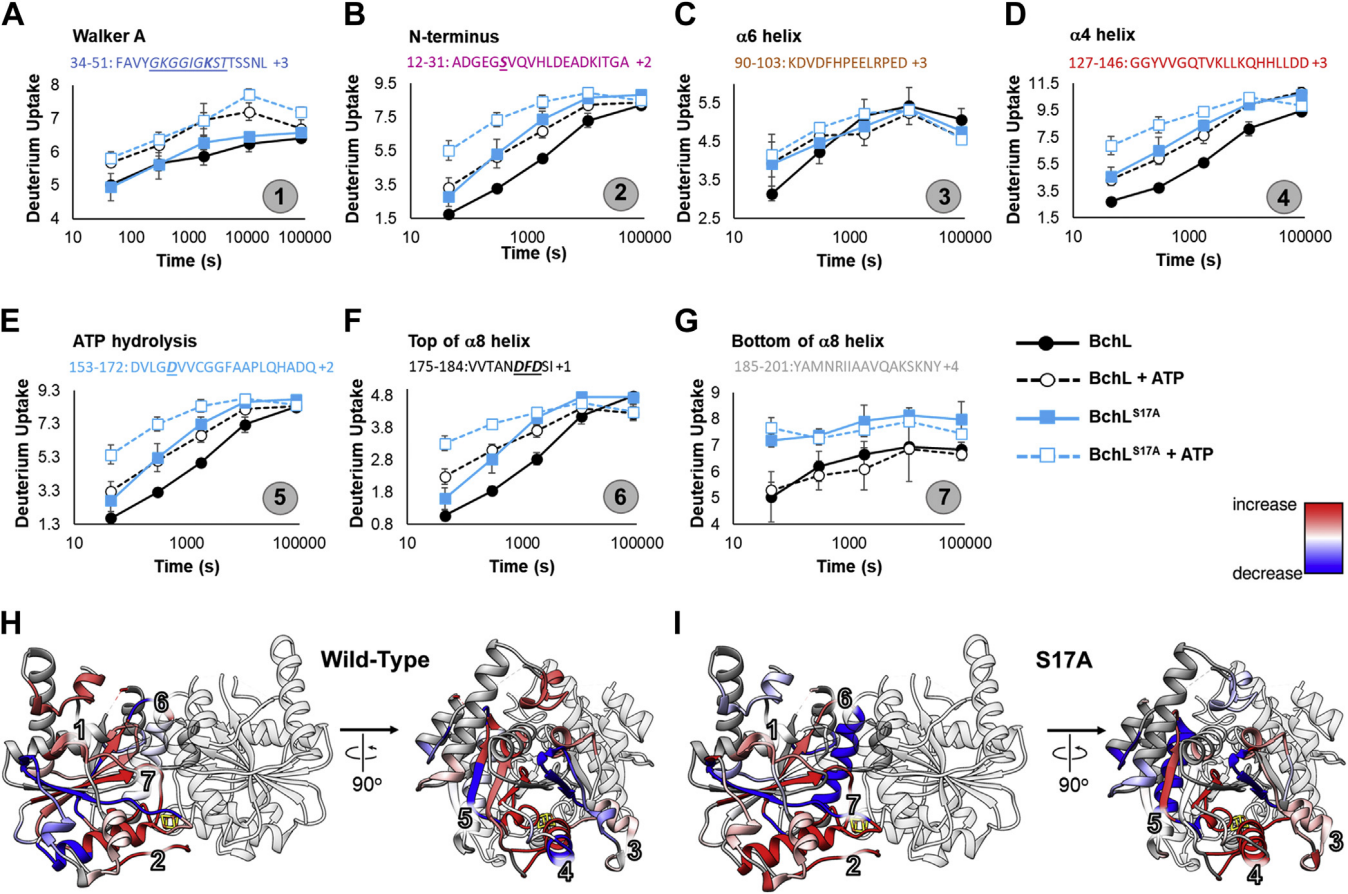
**Hydrogen–deuterium exchange mass spectrometry analysis of BchL and BchL**^**S17A**^**map out the ATP-driven conformation changes.***A–G*, the difference in deuteration levels observed for seven key regions (denoted by the circled number) from BchL and BchL^S17A^ in the presence or absence of ATP. Key residues in the Walker-A and Walker-B motifs, the flexible N-terminal tail, and the DFD patch are denoted (*underlined*) in the peptide sequence. The difference in deuteration levels observed for (*H*) WT BchL and (*I*) BchL^S17A^ is mapped onto the crystal structure. The seven key regions described in panels *A-G* are denoted. The scale of changes is colored *blue* (decrease in uptake) to *red* (increase in uptake). A decrease in uptake denotes shielding of the region from the solvent. Conversely, an increase in uptake reflects exposure of the region to the solvent.

We observe excellent sequence coverage maps for both BchL and BchL^S17A^ (Fig. S4). We identified 226 and 213 peptides for BchL and BchL^S17A^, respectively. This provided 96% and 88% coverage for BchL and BchL^S17A^, respectively. Multiple overlapping peptides were among these, providing exceptional coverage of nearly all regions of both proteins. A complete heat map of the HDX changes in the absence and presence of ATP is provided (Fig. S5). Here, we primarily focus on seven key regions that show robust changes in deuterium uptake or loss upon addition of ATP (Fig. 4). When these regions were highlighted on the crystal structure of BchL, it immediately highlighted a direct connection between the flexible N-terminal region and the nucleotide-binding pocket (Fig. 4, *H* and *I*, & Fig. S6).

Region 1 corresponds to peptides in the Walker-A motif (GXXXXGKST) that coordinates ATP binding (Fig. 4*A*). This peptide serves as an internal positive control as changes in this region should be observed upon ATP binding. Both BchL and BchL^S17A^ show similar patterns of reduced deuteration upon binding to ATP, reflecting shielding of the pocket from exchange. The similarity in the data also shows that nucleotide binding is not perturbed in BchL^S17A^. Region 2 includes peptides from the flexible N-terminal region (Fig. 4*B*). In the presence of ATP, both BchL and BchL^S17A^ show an increase in deuterium uptake, suggesting that this region becomes exposed upon nucleotide binding (Fig. 4*D*), in agreement with our EPR observations described earlier. The basal deuterium uptake is higher in BchL^S17A^ (Fig. 4*B*), in agreement with our prediction that that amino acid substitution (Ser-17 to Ala) should perturb this region based on contacts identified in our crystal structure (Fig. S1*B*).

Regions 3 and 4 include peptides from two α-helices (α6 and α4, respectively) that reside adjacent to the flexible N-terminus (Fig. 4, *C*–*D*) and share an extensive network of electrostatic and hydrophobic interactions (Fig. S7*A*). Both regions show an increase in deuteration in the presence of ATP, reflecting increased exposure to the solvent. Interestingly, in both these cases, the basal deuteration level of the BchL^S17A^ peptides align with the ATP-bound levels of BchL (Fig. 4, *C*–*D*). These data agree with our EPR observations that the electronic state of the [4Fe-4S] cluster in BchL^S17A^ closely resembles the ATP-bound conformation of BchL (Fig. 3*C*). Cys-126, which resides in helix α6 (region 4), also coordinates the [4Fe-4S] cluster. Taken together, these data support the model in which ATP-driven conformational changes lead to exposure of the metal cluster. Several residues in regions 3 and 4 are also part of the BchNB-binding interface (Fig. 2*C*), suggesting that ATP-driven perturbation of the contacts within this region and the flexible N-terminus lead to the release of autoinhibition.

The region 5 peptide contains Asp-157, a catalytic residue for ATP hydrolysis (Fig. 4*E*). Both proteins show an increase in deuteration upon binding to ATP, but BchL^S17A^ shows a markedly higher level of exposure to the solvent. Cys-160 is also located in this loop and coordinates the [4Fe-4S] cluster, and this region is directly connected to both α6 and α8 helices (Fig. S6, *B*–*C*). The HDX data suggest a direct path of communication between the N-terminal flexible region and the ATP-binding pocket. Perturbations in the N-terminal flexible region allosterically modulates changes in the ATP-binding pocket (and vice versa).

### A DFD amino acid patch promotes intersubunit cross stabilization upon ATP binding

**Figure 5:**
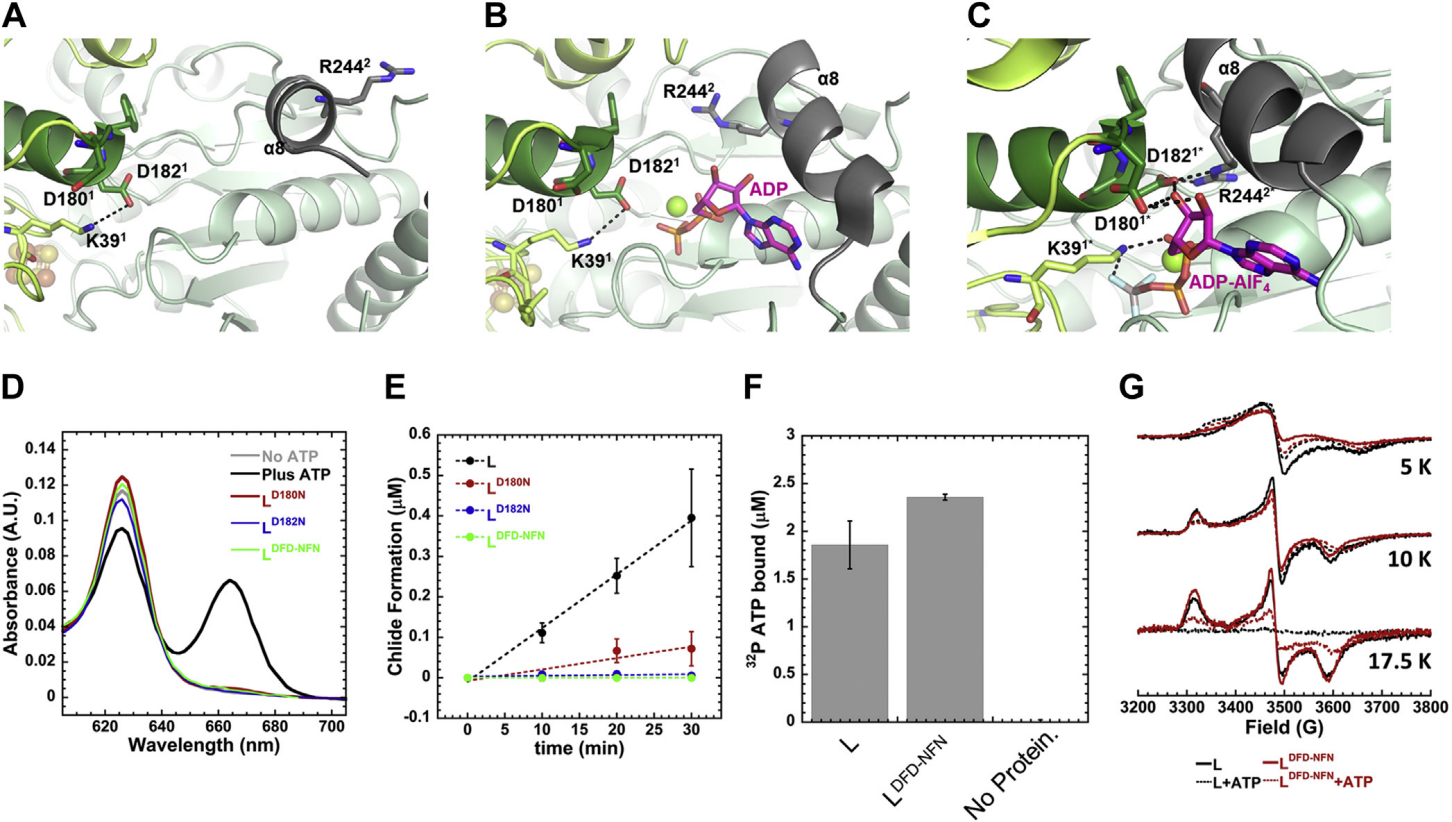
**Interactions between ATP and a DFD patch promote intersubunit cross stabilization and conformational changes in BchL.** The views of the DFD patch (highlighted as *green sticks*) and associated interactions in three distinct conformations of BchL. *A*, in the absence of nucleotides, residues Asp-180 and Asp-182 on chain D form no notable side-chain interactions and are far from residue Arg-244 on the opposing chain C. *B*, with ADP bound, the α8 helix, highlighted in *dark gray*, undergoes considerable motion, bringing Arg-244 closer to the DFD patch. However, no intersubunit interactions are observed. *C*, when in complex with ADP–AlF_3_ and BchNB, Asp-180 and Asp-182 have extensive interactions with both the bound nucleotide and intersubunit interactions with Arg-244. For clarity, residues are labeled in *Rhodobacter sphaeroides* numbering; the corresponding *Prochlorococcus marinus* residues are Asp-178, Asp-180, and Arg-242. *D*, Pchlide reduction activity of BchL and variant proteins collected 60 min after addition of ATP. Chlide formation is monitored as a peak between 660 and 670 nm and occurs only in the presence of ATP. BchL^D180N^, BchL^D182N^, and BchL^DFD-NFN^ are all defective for substrate reduction. *E*, time course of Chlide formation for reactions containing BchNB, ATP, and Pchlide in the presence of various BchL constructs. BchL reduces Pchlide to Chlide (*k*_*obs*_ = 0.0126 ± 0065 μM·min^-1^), and no appreciable Chlide formation is observed for BchL^D182N^ or BchL^DFD-NFN^. Severely impaired but detectable reduction activity is observed for BchL^D180N^ (*k*_*obs*_ = 0.0028 ± 0.0042 μM·min^-1^). *F,* nitrocellulose filter binding analysis of ATP binding to BchL shows ∼2 ATP bound per BchL dimer to both BchL and BchL^DFD-NFN^, and no nonspecific binding to the membranes is observed in the absence of BchL in the reaction. *G*, EPR spectra comparing BchL (*black solid lines*), BchL incubated with excess ATP (*black dotted lines*), BchL^DFD-NFN^ (*red solid lines*), and BchL^DFD-NFN^ incubated with excess ATP (*red dotted lines*) at 5 K, 10 K, and 17.5 K as denoted. Chlide, chlorophyllide; Pchlide, protochlorophyllide.

The ATP-binding sites in BchL are situated away from the BchL–BchNB interaction interface (Fig. 1*A*, Fig. 2, *A*–*C*), but the protein must somehow be able to communicate the nucleotide-binding state to the cluster and tail regions. The long α-helix (α8; Fig. S6*B*) connects the top and bottom halves on BchL and might function as a conduit for communication relaying ATP binding–driven changes to promote rearrangement of the flexible N-terminus. Regions 6 and 7 contain peptides from the top and bottom halves of this helix (Fig. 4, *F*–*G*). Region 7 shows the largest HDX difference between BchL and BchL^S17A^, with the variant protein showing an increase in deuteration (Fig. 4*G*). Region 6, the top half of the helix, shows an ATP-dependent increase in deuteration with higher levels of exposure observed for BchL^S17A^. Comparison of the nucleotide-free (Fig. 5*A*), ADP-bound (Fig. 5*B*) (10), and ADP-AlF_3_–NB–bound (Fig. 5*C*) (11) L-protein crystal structures reveal a ‘DFD patch’ at the top part of this helix composed of conserved amino acid residues Asp-180, Phe-181, and Asp-182 that undergo rearrangements and contact the hydroxyl groups of the sugar moiety of the bound nucleotide. In the ADP-AlF_3_–NB–bound complex structure, Asp-180 and Asp-182 (*R. sphaeroides* numbering; Asp-178 and Asp-180 in *P. marinus*) from one subunit form a network of interactions in *trans* with the sugar moiety of the nucleotide bound to the neighboring subunit along with Arg-244 in the nucleotide-bound subunit (Fig. 5*C*). We have termed this series of interactions “intersubunit cross stabilization of ATP”.

Although ATP-driven intersubunit cross stabilization within the Fe protein is also present in the homologous nitrogenase system (23), interactions involving the DFD patch region appear to be specific to BchL (Fig. S7). In the both the ADP-bound structure and our nucleotide-free structure, Asp-182 appears to form a weak hydrogen bond with Lys-39 of the same chain but does not interact with the other subunit (Fig. 5, *A*–*B*). However, in the ADP–AlF_3_–NB–bound L-protein structure, Lys-39 and Asp-182 do not interact and instead form significant interactions with the bound nucleotide (Fig. 5*C*). Asp-182 additionally forms an intersubunit interaction with Arg-244 of the opposite chain. In nitrogenase, these interactions are not conserved (Fig. S8) as the analogous residue to Asp-182 is instead hydrophobic, Met-156 in *Azotobacter vinelandii* nitrogenase (Fig. S8*B*) and cannot participate in electrostatic interactions with the opposing chain. Overall, although the phenomenon of ATP-dependent intersubunit cross stabilization appears to be common to the two systems, the precise mechanisms are somewhat different.

As these aforementioned contacts in DPOR only appear in the ADP–AlF_3_–L–NB structure (Fig. 5*C*, and Fig. S7), the DFD patch appears poised to be important for coordinating ATP-dependent conformational changes during complex formation and Pchlide reduction. Based on the HDX changes in this region (Fig. 4*F*), we hypothesized that ATP binding permits intersubunit cross stabilization and would provide the necessary conformational stability required to pry away the flexible N-terminus from binding across the [4Fe-4S] cluster, thus relieving inhibition. If this were the case, amino acid substitutions in the DFD patch would not affect ATP binding but would perturb Pchlide reduction.

To test this hypothesis and the role of the DFD patch, we generated three BchL variants carrying amino acid substitutions wherein Asp-180 (BchL^D180N^), Asp-182 (BchL^D182N^), or both (BchL^DFD-NFN^) were substituted to Asn. Phe-181 does not make contacts with the bound nucleotide and thus was not perturbed. BchL^D180N^ was poorly active for Pchlide reduction, whereas BchL^D182N^ and BchL^DFD-NFN^ were inactive (Fig. 5, *D*–*E*), suggesting that both residues are important for function. Next, to precisely understand why amino acid substitutions in the DFD patch affected Pchlide reduction, we analyzed the nucleotide binding and EPR spectral properties of BchL^DFD-NFN^. Nucleotide binding was measured by capturing the BchL–ATP complex using radiolabeled α^32^P-ATP in a nitrocellulose filter binding assay. BchL binds to the nitrocellulose filter, and ATP bound to the protein is retained on the membrane, whereas unbound ATP flows through the filter. Both BchL and BchL^DFD-DFN^ are capable of binding to ATP, and no ATP is retained on the membrane when no protein is present in the reaction (Fig. 5*F*). This finding is consistent with the ADP-bound crystal structure where no contacts between the DFD patch and nucleotide are observed (Fig. 5*B*). Thus, the loss in Pchlide reduction activity in the BchL^DFD-NFN^ protein occurs after ATP binding and is possibly due to a loss of the promotion of conformational changes necessary for multiple rounds of complex formation with BchNB.

EPR of BchL^DFD-NFN^ at 5 K (Fig. S3*E* and Fig. 5*G*) again revealed an FeS^A^ signal, although the rapid-passage distortions indicated that ATP binding enhances relaxation in BchL^DFD-NFN^ and ATP inhibits relaxation in WT BchL. The overall relaxation rates for FeS^A^ in WT BchL and BchL^DFD-NFN^ can be summarized as BchL > BchL-ATP ≈ BchL^DFD-NFN^-ATP > BchL^DFD-NFN^. At 10 K, the EPR spectra of both WT BchL and BchL^DFD-NFN^ are almost indistinguishable and consist of 60% FeS^B^, whereas the ATP complexes of both exhibit EPR signals indistinguishable from each other but containing only 35% FeS^B^ and 65% of the relaxation-inhibited FeS^A^ (Fig. 5*G* and Fig. S3*E*). The spectra of both WT BchL and BchL^DFD-NFN^ at 17.5 K are again indistinguishable and are due to FeS^B^ alone (Fig. S3*E*). In both cases, the signals are of diminished intensity upon addition of ATP because of enhanced relaxation, to about 25% in the case of BchL^DFD-NFN^-ATP and almost completely extinguished in WT BchL-ATP (Fig. 5*G*). It is not clear whether the residual FeS^B^ signal from BchL^DFD-NFN^-ATP at 17.5 K is due to slower relaxation than in WT BchL-ATP or less than stoichiometric binding of ATP. Overall, relaxation of FeS^A^ is markedly inhibited in BchL^DFD-NFN^, indicating poorer coupling to a strained lattice in the species correlated with the nucleotide-free species in the WT context, whereas the cluster environments in the FeS^B^ species of WT BchL and BchL^DFD-NFN^ are indistinguishable by EPR (Fig. 5*G*, 17.5 K *red* and *black solid traces*), and the binding of ATP to both species provides very similar cluster environments for both FeS^A^ and FeS^B^. Thus, ATP binding to BchL^DFD-NFN^ does not elicit the complete portfolio of conformational changes required for Pchlide reduction.

### Binding of ATP to both subunits in the BchL dimer is required to generate a concerted motion to promote intersubunit cross stabilization and drive Pchlide reduction

**Figure 6:**
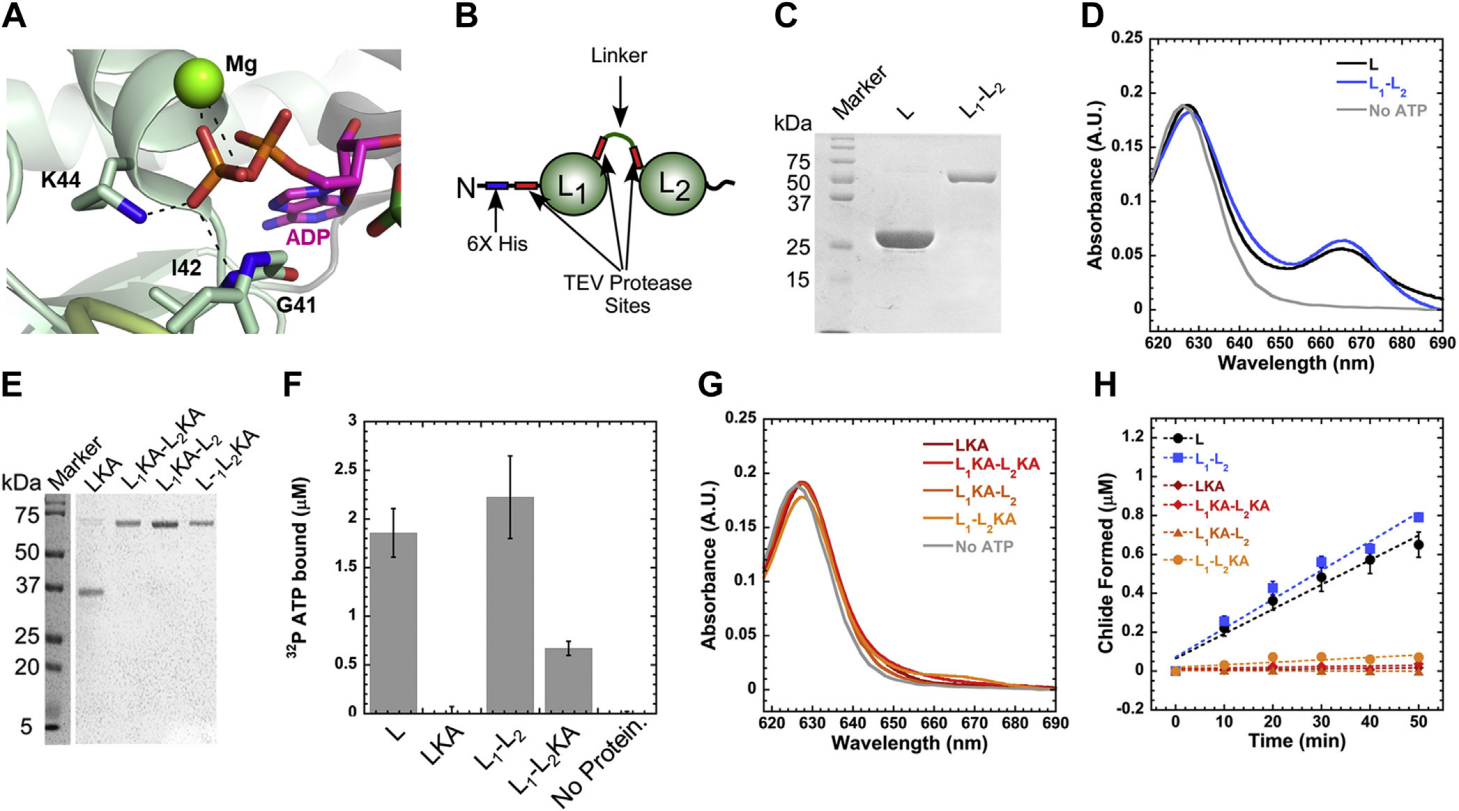
**Both ATP binding sites are required for DPOR function.***A*, Lys-44 stabilizes nucleotide binding through interactions with the phosphate group of ADP (PDB ID: 3FWY). *B*, the schematic of the linked-L-protein design and the positions of the TEV protease cleavage sites. *C*, SDS-PAGE analysis of the purified BchL and linked-BchL-proteins. *D*, spectroscopic analysis of Pchlide reduction activity of BchL (L, *black trace*), linked-BchL-protein (L1-L2, *blue trace*), and no-ATP negative control (no ATP, *gray trace*). Pchlide absorbance is observed at 625 nm, and Chlide formation is monitored at 665 nm 60 min after incubation with ATP. *E*, SDS-PAGE analysis of the purified BchL^K44A^ (LKA) and versions of the single and double Lys-44 to Ala–substituted linked-BchL-proteins. *F,* nitrocellulose filter binding analysis of ^32^P-ATP binding by WT and Lys-44 to Ala–substituted BchL proteins. L and L1-L2 are capable of binding ATP, whereas the singly substituted L_1_KA-L_2_ is partially able to bind ATP. When both subunits are substituted with Lys44 to Ala, no ATP binding is observed. *G*, linked and unlinked BchL proteins carrying the K44A substitution are incapable of reducing Pchlide. Data shown were collected 60 min after incubation with ATP. *H*, kinetics of Pchlide reduction measured as a function of Chlide formation is shown. L and L_1_-L_2_ reduce Pchlide to Chlide (*k*_*obs*_ = 0.01266 ± 0.007 μM·min^-1^ and 0.0148 ± 0.0022 μM·min^−1^, respectively). DPOR, dark-operative protochlorophyllide oxidoreductase; Chlide, chlorophyllide; Pchlide, protochlorophyllide; TEV, tobacco etch virus.

Each BchL homodimer consists of two sites for ATP binding, and the two subunits are covalently tethered by a single [4Fe-4S] cluster (Fig. 2, *A*–*C*). In the homologous Fe-protein of nitrogenase, the sites have been shown to bind to nucleotides with differing affinities (24). A crystal structure of the Fe-protein with two different nucleotides occupying the dimer has also been solved, suggesting that the two ATP sites could play distinct roles in dinitrogen reduction (25) (Fig. S7*C*). To test the functional role of the two ATP-binding sites in BchL, we generated a covalently linked version of BchL by expressing the two subunits as a single polypeptide. The "linked” BchL construct included the same N-terminal 6x-poly histidine tag followed by a tobacco etch virus protease site identical to all other L-protein constructs. Two BchL subunits were tethered by a flexible linker that connected the C-terminal end of the first monomer to the N-terminal end of the other (Fig. S9*A*). Covalent linkage of multimers can interfere with the activity because of a variety of effects including conformational strain, nonspecific interactions due to the linker, introduction of non-native secondary structures, or undefined effects. The linker was therefore constructed to carry tobacco etch virus protease recognition sites bookending the linker region, enabling proteolytic removal if the intact linker interfered with the activity for any reason. We found that the length of the linker is a key determinant of protein activity. Linkers shorter than 15 amino acids are defective for Pchlide reduction, and the activity similar to WT BchL is obtained when linker lengths are longer than 20 amino acids (Fig. S9, *C*–*D*). The optimized linked-BchL behaved similarly to WT BchL during purification (Fig. 6*C*) and was stable and fully active for Pchlide reduction (Fig. 6*D*). Removal of the linker after protease cleavage also resulted in protein activity similar to the uncleaved and WT BchL proteins (Fig. S9*B*). These results show that a linker of an optimal length does not interfere with protein function. The linked L-protein appears to have slightly faster Pchlide reduction activity (*k*_*obs*_ = 0.01266 ± 0.007 μM·min^-1^ and 0.0148 ± 0.0022 μM·min^-1^, respectively; Fig. 6*H*). The rate of Pchlide reduction for the linked L-protein resides between those of the WT BchL and BchL^S17A^. In the case of WT BchL and BchL^S17A^, there are two available tails (one from each BchL monomer), one of which can bind across the NB-protein binding interface. Owing to the pseudo-C2 symmetry, at any given point, one of the two tails can autoinhibit DPOR activity. In the BchL^S17A^ variant, neither N-terminus can serve the autoinhibitory role, leading to an increased rate of Pchlide reduction. The linked-BchL variant, on the other hand, only has one free N-terminus as the other is covalently tethered to the next BchL subunit. Thus, the NB-protein docking interface may be slightly more accessible than that of WT BchL, leading to higher rates of Pchlide reduction (Fig. 6*H*).

Next, to assess the contribution of the individual ATP sites, we generated an ATP-binding–deficient amino acid substitution in one or both subunits in linked BchL (Fig. 6*E*). Substitution of the conserved Lys-44 with an Ala in the Walker-A motif is expected to perturb ATP binding (Fig. 6*A*). Using filter-binding analysis, we determined the amount of ATP bound to the linked and unlinked versions of BchL. BchL (L) and linked-BchL with unaltered ATP-binding sites (L_1_L_2_) bind ATP to similar extents (Fig. 6*F*). The Lys-44 to Ala substitution in the Walker-A ATP-binding pocket of BchL (L^KA^) and in both subunits of the linked-BchL (L_1_^KA^-L_2_^KA^) abolishes ATP binding as expected (Fig. 6*F*). When only one of the two ATP binding sites is mutated (L_1_^KA^-L_2_), partial ATP binding is observed (Fig. 6*F*). When ATP binding is perturbed in both sites, or in just one site, a complete loss of Pchlide reduction activity is observed (Fig. 6, *G*–*H*). These data suggest that both ATP molecules in the BchL dimer are required for Pchlide reduction. Based on these data, we propose that binding of both ATP molecules likely causes cooperative conformational changes in the two halves of the L-protein homodimer and drives intersubunit cross stabilization.

## Discussion

Nitrogenase and nitrogenase-like enzymes such as DPOR and COR share structural similarity with respect to their electron donor and electron acceptor component proteins. These proteins catalyze multiple rounds of ET for substrate reduction, and transient association of the electron donor and electron acceptor is a prerequisite for each ET event. ATP binding to the electron donor is canonically assigned as the mechanistic trigger that promotes the assembly of the component proteins. However, the precise structural and functional principles underlying this ATP-driven process have largely remained unclear. The results presented in this study shed light on several ATP binding–driven changes in BchL that represent previously undiscovered allosteric regulatory mechanisms.

In our nucleotide-free crystal structure, residues 16 to 29 are ordered in one of the four chains and suggest a novel regulatory role for the flexible N-terminus of BchL. Interestingly, the N-terminus binds across the [4Fe-4S] cluster and interacts both directly and indirectly with residues involved in docking with BchNB (Fig. 2, *A*–*C*). Amino acid substitutions that perturb specific interactions in this region enhance Pchlide reduction activity, supporting an autoinhibitory role for this region in DPOR function (Fig. 3). Although binding of ATP can be thought to trigger conformational changes leading to the displacement of N-terminal residues, only ATP hydrolysis drives the dissociation of the BchL-NB complex to complete the catalytic cycle. Therefore, the conformational change in the N-terminal region could be yet another way of coupling ATP binding to BchL with Pchlide reduction in BchNB.

**Figure 7:**
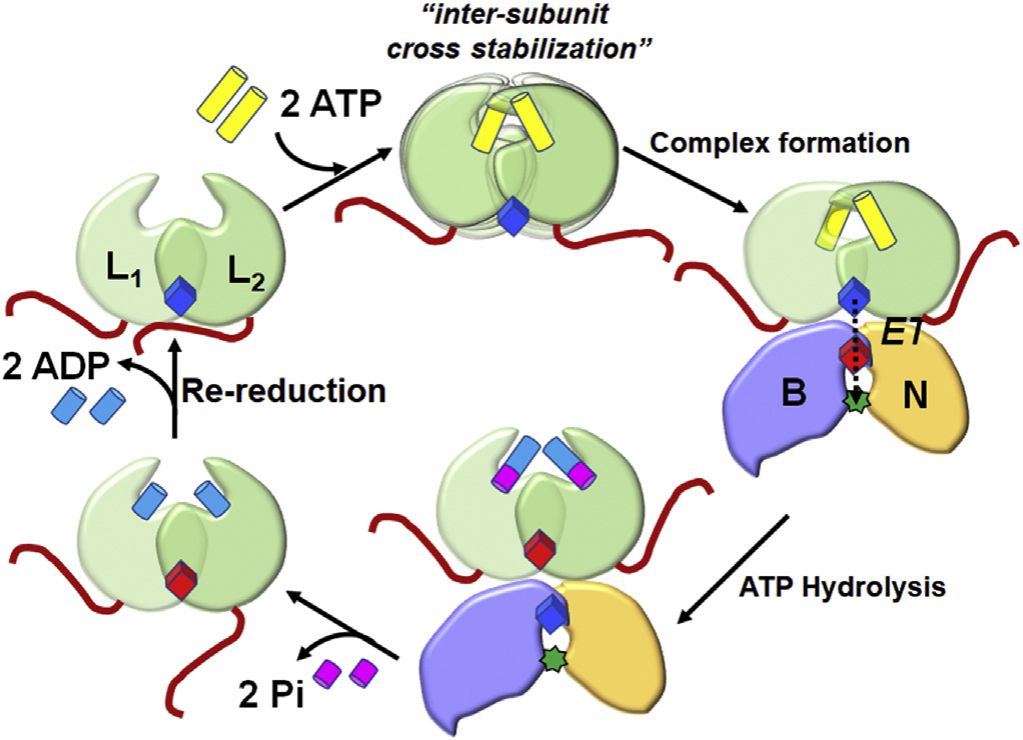
**Model for ATP binding–induced release of autoinhibition by the flexible N-terminus of BchL.** Cartoons depict the BchL dimer (*green*) with one of the two disordered N-terminal flexible regions binding across the [4Fe-4S] cluster (*blue*; reduced form of the cluster). ATP (*yellow*) binding promotes intersubunit cross stabilization and associated conformational changes through interactions between the DFD patch and ATP, thereby releasing the flexible N-terminus from the docking surface. Complex formation to the BchNB protein ensues followed by electron transfer (ET), ATP hydrolysis, and product (ADP, Pi) release. The oxidized BchL [4Fe-4S] cluster is depicted in *red*. The BchL [4Fe-4S] cluster is subsequently re-reduced by ferredoxin. The *green star* represents Pchlide (substrate) bound to the active site of BchNB. Chlide, chlorophyllide; Pchlide, protochlorophyllide.

ATP binding to both subunits of BchL promotes a network of interactions between a conserved DFD patch and the sugar moiety of the nucleotide, which in turn stabilizes intermolecular interactions across subunits. Such an ATP binding–dependent conformational change could generate an upward compaction of BchL, leading to the release of the N-terminus from the docking interface and promoting complex formation with BchNB (Fig. 7). Substitutions in the DFD patch and rendering one subunit devoid for ATP binding are both able to abolish Pchlide reduction activity. We thus propose a model where cooperative interactions between the DFD patch and the nucleotide relieves the autoinhibition by the N-terminus of BchL and is a key regulatory step in transient assembly of the DPOR complex (Fig. 7).

The HDX-MS data support an elegant allosteric mechanism for the ATP binding–driven release of autoinhibition, as helix α8 physically connects the DFD patch and the flexible N-terminal region (Fig. S6, *B*–*C*). ATP binding may serve to pull the top of the α8 helix (DFD patch) from each subunit, causing a compaction at the top of the BchL homodimer. This in turn would result in a relaxation of the contacts at the bottom of the BchL homodimer, thus prying away the flexible N-terminal region (Fig. 7). Similarly, interactions between helix α6 and α4 would also be affected (Fig. S6*A*) and since one end of α6 coordinates the [4Fe-4S] cluster, the cluster’s local environment and therefore its electronic properties would be affected. Such a change is further supported by in our EPR measurements. Finally, both the Walker-A (P-loop; Lys-44) and the Walker-B loops carrying the catalytic residue (Asp-157) for ATP hydrolysis are connected to α6 and α8 (Fig. S6*C*) and complete the allosteric network of interactions in BchL.

In the related nitrogenase complex, several crystal structures of the homologous Fe-protein have been solved in complex with a variety of nucleotides in the absence and presence of the MoFe-protein. In these structures, the relative distances between the two nucleotides do not change significantly (Fig. S7). Because the flexible N-terminal region is not conserved in the Fe-protein of nitrogenase, it may indicate a difference in the mechanism between the DPOR and nitrogenase systems.

In DPOR, the structural changes observed between the ADP-bound and the nucleotide-free crystal structures additionally suggest how nucleotide binding may enable ET from BchL to BchNB. Comparison of the two structures suggests that switch II region (residues 153–164) may act as a redox switch, analogous to its function in the Fe-protein of nitrogenase where it is thought to communicate changes in the nucleotide-binding site to the [4Fe-4S] cluster environment (23, 26, 27, 28). The switch II region is fully conserved between BchL and NifH (the nitrogenase Fe-protein), including Phe-135 in NifH (Phe163 in BchL), which has been demonstrated to be important for the redox properties of the [4Fe-4S] cluster of NifH (27, 28). In the absence of ADP, the local environment immediately surrounding the BchL cluster is more packed and hydrophobic, promoted by the interactions of Leu-155, Val-158, and Phe-163 (Fig. S7). Although crystal packing effects can complicate interpretation, a similar pattern is generally observed when comparing substrate-free and ADP-bound nitrogenase Fe-protein structures (26, 27, 29, 30, 31). In general, for redox-active metal centers, hydrophobicity increases reduction potential (32, 33), suggesting that in the absence of Mg-ADP, the BchL cluster is less likely to become oxidized. Although electrochemical data are not available for BchL, studies with NifH show that in the presence of nucleotides, the midpoint reduction potential is 120 mV (for ATP) or 160 mV (for ADP) more negative (28), indicating that oxidation of the [4Fe-4S] cluster of NifH for ET to the P-cluster becomes more favorable in the presence of nucleotides. The conservation of switch II sequence suggests that the redox properties of the [4Fe-4S] cluster of BchL could be similarly modulated. The van der Waals interaction observed between the [4Fe-4S] cluster of BchL, its ligand Cys-160, and Phe-163 (Fig. 2*C* and Fig. S10) could be a means of modulating the redox properties of the BchL-[4Fe-4S] cluster in the presence and absence of nucleotides.

A BLAST-P analysis of residues 1 to 29 yielded BchL protein hits spread across ∼150 species of eubacteria. A nine-residue sequence in the flexible N-terminus [DGEGSVQVH] (residues 13–21 in *R. sphaeroides* BchL) is highly conserved, supporting functional significance (Fig. 2*E*). The flexible N-terminal region is unique to the eubacterial DPOR system. DPOR of cyanobacteria and chloroplasts of higher plants use ChlL, a functional homolog of BchL. The flexible N-terminal region is not conserved in ChlL. However, ChlL possesses a unique disordered C-terminal tail (Fig. S11). Whether this region plays a regulatory role in ChlL function remains to be investigated. One exception to this evolutionary pattern is found in the cyanobacterium *P. marinus* where its ChlL protein resembles BchL. The crystal structures L-protein from *R. sphaeroides* and *P. marinus* are similar (PDB IDs: 3FWY and 2YNM; Fig. 2, *B*–*C*), and both proteins possess the flexible N-terminal region. This region does not show strong conservation in BchX of eubacterial COR that catalyzes the subsequent reductive step in bacteriochlorophyll synthesis (Fig. S2), and such an N-terminal region does not exist in the Fe-protein of nitrogenase (Fig. S2*A*).

Higher plants and cyanobacteria differ from photosynthetic eubacteria as they have acquired an oxygen-evolving complex; the aerobic environment within the chloroplast is significantly different than that of anaerobic eubacterial cells. It is possible that the differences in the flexible N-terminal regions may reflect responses to the evolutionary pressures of an aerobic environment. The biological necessity for such a regulatory region remains to be established, although may exist as a mechanism to prevent off-target electron donation and the formation of free radicals in the cell.

## Experimental procedures

### Reagents and buffers

Chemicals were purchased from Sigma-Millipore Inc (St Louis, MO), Research Products International Inc (Mount Prospect, IL), and Gold Biotechnology Inc (St Louis, MO). Oligonucleotides for cloning were purchased from Integrated DNA Technologies (Coralville, IA). Enzymes for molecular biology were purchased from New England Biolabs (Ipswich, MA). All reagents and buffers were thoroughly degassed using alternating cycles of vacuum and nitrogen pressure on a home-built Schlenk line apparatus. Anaerobic conditions were maintained *via* airtight syringes, excess reductant (dithionite), and a vinyl glove box (Coy Laboratories, MI) under a nitrogen (95%) and hydrogen (5%) mix atmosphere.

### Generation of protein overexpression constructs

The coding regions for BchL, BchN, and BchB were PCR-amplified from *R. sphaeroides* genomic DNA (a kind gift from Dr Scott Ensign, Utah State University) and cloned into pRSF-Duet 1 or pET-Duet 1 plasmids as described (34). Amino acid substitutions in BchL were generated using Q5 site-directed mutagenesis (New England Biolabs). Plasmids used to express the linked-BchL-proteins carrying glycine linkers of various lengths were synthesized as codon-optimized genes (GenScript Inc, Piscataway, NJ). The longest iteration of the linked-BchL-protein was generated as described in the supplemental section. The difference in the sequences between the linked-BchL with the longest linker (L21) and the shorter linkers arose because of the differing cloning versus synthetic strategies used to generate the constructs (Fig. S9).

### Protein purification

BchL and BchNB proteins were overexpressed and purified as originally described (35), with modifications as recently reported (34). The following additional steps were added to the purification of the linked-L-proteins. During cell lysis and all subsequent steps, protease inhibitors (protease inhibitor cocktail, MilliporeSigma Inc—catalog no. P2714) and 1-mM PMSF were added to all buffers. As an additional purification step, the concentrated linked-L-protein from the Q-Sepharose eluate was subsequently fractionated over a Sephadex S200 26/600 PG (GE Life Sciences) column using standard buffer (100-mM Hepes, pH 7.5, 150-mM NaCl, 10-mM MgCl_2_, 1.7-mM sodium dithionite, 1-mM PMSF, and protease inhibitors). Protein concentrations were determined using the Bradford assay and bovine serum albumin as the standard.

### Generation of Pchlide

Pchlide was generated from a *Rhodobacter capsulatus* ZY-5 strain harboring a deletion of the BchL gene (a kind gift from Dr Carl Bauer, Indiana University) (36) and purified as described (34).

### Pchlide reduction assays

Reduction of Pchlide to Chlide was measured spectroscopically by mixing BchNB (5-μM tetramer), BchL (20-μM dimer), and 35-μM Pchlide, in the absence or presence of ATP (3 mM) in standard buffer with 10-mM MgCl_2_. Pchlide reduction experiments were carried out in 200-μl reactions and quenched at the denoted time points with 800 μl of 100% acetone. The acetone/reaction mixture was spun down in a table-top centrifuge at 13,226 x *g* for 4 min. The supernatant was transferred to a cuvette and absorbance scans from 600 nm to 725 nm were recorded on a Cary 100 UV-Vis spectrophotometer (Agilent Technologies). Chlide appearance was quantified using the molar extinction coefficient 74,900 M^-1^ cm^-1^ at 666 nm. To observe Pchlide reduction in real time (data shown in Fig. 3), Chlide appearance was measured in the aqueous solution (instead of quenching with acetone at the denoted time points) using a Type-41 macrocuvette with a screw cap (Firefly Scientific, Staten Island, NY). Reactions contained BchNB (1-μM tetramer), BchL (4-μM dimer), 35-μM Pchlide, and ATP (3 mM) in standard buffer containing 10-mM MgCl_2_. Reactions were initiated by the addition of degassed ATP *via* a gas-tight syringe, and spectra were recorded from 400 to 800 nm every 60 s as described above. The Pchlide reduction traces were normalized using spectra recorded before ATP addition.

### BchL crystallization

BchL crystals were grown anaerobically (100% N_2_ environment with <0.1 ppm O_2_) inside a Unilab Pro glove box (mBraun, Stratham, NH) at 15 °C using the vapor diffusion method (37). All materials and buffers were pretreated to remove oxygen as previously described (37). Initial sparse matrix screens were set up anaerobically using a Mosquito crystal robotic liquid handler (TTP Labtech, Boston, MA). 1 μl of 200-mM sodium dithionite solution was added to every well (90 μl) to ensure complete removal of any dissolved oxygen. For each drop, 200 nl of the well solution and 200 nl of 100-μM dimeric BchL (in 100-mM Hepes, pH 7.5, 150-mM NaCl, 10% (v/v) glycerol, 1.7-mM dithionite) were mixed. A crystal was observed after approximately 1 month with a well solution consisting of 0.6 M sodium chloride, 0.1 M MES:NaOH, pH 6.5, 20% (w/v) PEG 4000. Larger volume (3–4 μl) drops of the same well solution and protein concentration in 1:1, 1:2, and 2:1 ratios of protein to the well solution yielded large, dark brown single crystals after ∼2 to 3 months. Before freezing, the well solution was mixed in an equal volume of cryoprotectant solution with a final concentration of 9% sucrose (w/v), 2% glucose (w/v), 8% glycerol (v/v), and 8% ethylene glycol (v/v). Crystals were soaked for a few seconds in the cryoprotectant before being cryocooled in liquid nitrogen. A crystal from a drop set up with 2-μl well solution and 2-μl protein solution was used for in-house data collection, whereas different crystals that grew with 2-μl well solution and 1-μl protein solution were used for synchrotron data collection.

### BchL data collection, processing, and refinement

An initial model was built using data collected with an in-house Rigaku MicroMax-007 HF X-ray source equipped with a Pilatus 300K detector. A complete data set at cryogenic temperature (100K) was collected to 2.92-Å resolution, which was integrated using HKL2000 (38) and merged and scaled using SCALA in the CCP4 suite (39). Phase determination was initially estimated through molecular replacement (PHASER) using the *R. sphaeroides* ADP-bound BchL structure (PDB ID: 3FWY, with all ligands removed) as the search model (10, 40). A solution was found with two dimers in the asymmetric unit. Following molecular replacement, rigid-body refinement was performed in Phenix (40). A starting model was built using AutoBuild and further improved with iterative rounds of model building and refinement using COOT (41) and Phenix.

Higher resolution data were collected on different crystals at beamline 17-ID-1 highly automated macromolecular crystallography, the National Synchrotron Light Source-II, at the Brookhaven National Laboratory on a Dectris Eiger 9M detector. Two complete data sets collected at 100 K were integrated, scaled, and merged to 2.6-Å resolution using HKL2000. The partially refined model from home-source data was used as a molecular replacement model for solving the structure in Phenix. The resulting model was improved through iterative rounds of model building using COOT and Phenix. Data processing and refinement statistics are presented in Table S1.

### EPR spectroscopy

EPR spectra were obtained at 5, 10, 17.5, and 30 K on an updated Bruker EMX-AA-TDU/L spectrometer equipped with an ER4112 SHQ resonator (9.48 GHz) and an HP 5350B microwave counter for precise frequency measurement. The temperature was maintained with a ColdEdge/Bruker Stinger S5-L recirculating-helium refrigerator and an Oxford ESR900 cryostat and a MercuryiTC temperature controller. Spectra were recorded with either 0.3 G (3 × 10^-5^ T) or 1.2 G (0.12 mT) digital field resolution with equal values for the conversion time and the time constant, 1.0-mW incident microwave power, and 12 G (1.2 mT) magnetic field modulation at 100 kHz. EPR simulations were carried out using EasySpin (42).

### Samples for EPR spectroscopy

200 μl EPR samples contained 40-μM BchL (or variant), 1.7-mM dithionite, and, where indicated, 3-mM ATP, 3-mM ADP, and/or 20-μM BchNB. A subset of the EPR experiments were carried out with 20-μM BchNB, 40-μM Pchlide, and, where indicated, 3-mM ATP. Protein samples were prepared and transferred to the EPR tubes in the glove box and stoppered with a butyl rubber stopper. Samples were removed from the glove box and immediately flash-frozen in liquid nitrogen and then analyzed by EPR.

### HDX-MS

Anaerobically purified BchL and BchL^S17A^ stocks (23 mg/ml and 21 mg/ml, respectively) were diluted 1:10 into an anaerobic deuterated reaction buffer (100-mM Hepes, 150-mM NaCl, 10-mM MgCl_2,_ 1.7-mM sodium dithionite, pD 7.5 in D_2_O) in the presence and absence of 2-mM ATP_._ Anaerobic conditions were maintained as described (43). Anaerobic control samples were diluted into a nondeuterated reaction buffer. At each time point (0, 45, 300, 1800, 10,800, and 86,400 s), 10 μl of the reaction buffer was removed from a crimp-sealed reaction vial using a gas-tight syringe and quenched by adding it to 60 μl of 0.75% formic acid (FA, Sigma) and 0.25 mg/ml porcine pepsin (Sigma) at pH 2.5 on ice. Each sample was digested for 2 min with vortexing every 30 s and then flash-frozen in liquid nitrogen. Samples were stored in liquid nitrogen until the LC-MS analysis. The LC-MS analysis of BchL was completed as described (44). Briefly, the LC-MS analysis of BchL was completed on a 1290 UPLC series chromatography stack (Agilent Technologies) coupled with a 6538 UHD Accurate-Mass QTOF LC/MS mass spectrometer (Agilent Technologies). Peptides were separated on a reverse phase column (Phenomenex Onyx Monolithic C18 column, 100 x 2 mm) at 1 °C using a flow rate of 500 μl/min under the following conditions: 1.0 min, 5% B; 1.0 to 9.0 min, 5 to 45% B; 9.0 to 11.8 min, 45 to 95% B; 11.8 to 12.0 min, 5% B; solvent A = 0.1 % FA (Sigma) in water (Thermo Fisher) and solvent B = 0.1% FA in acetonitrile (Thermo Fisher). Data were acquired at 2 Hz s^-1^ over the scan range 50 to 1700 m/z in the positive mode. Electrospray settings were as follows: the nebulizer set to 3.7 bar, drying gas at 8.0 L/min, drying temperature at 350 °C, and capillary voltage at 3.5 kV. Peptides were identified as previously described (45) using MassHunter Qualitative Analysis, version 6.0 (Agilent Technologies), Peptide Analysis Worksheet (ProteoMetrics LLC), and PeptideShaker, version 1.16.42, paired with SearchGUI, version 3.3.16 (CompOmics). Deuterium uptake was determined and manually confirmed using HDExaminer, version 2.5.1 (Sierra Analytics). Heat maps were created using MSTools (46).

### ATP-binding assay

Nitrocellulose membranes, cut into 2- × 2-cm squares, were pretreated with 0.5 N NaOH for 2 min, washed extensively with H_2_O, and equilibrated in the binding buffer (100-mM Hepes, pH 7.5, 150-mM NaCl, and 10-mM MgCl_2_). In the reactions (100 μl), BchL (4 μM) was incubated with 1-mM ATP +0.3-μCi α^32^P-ATP for 10 min at 25 ºC, and 20-μl aliquots of the reaction mixture were filtered through the membrane on a single filter holder (VWR Scientific Products). The membranes were washed before and after filtration with 250 μl of the nucleotide-binding buffer and air-dried before overnight exposure onto a PhoshorImaging screen. 1-μl aliquots were spotted onto a separate membrane to measure the total nucleotide in the reaction. Radioactivity on the membrane was quantitated on a PhosphorImager (GE Life Sciences). Total ^32^P-ATP bound was calculated using the following equation: [[bound^signal^]/[[total^signal^] x 20]]] x [ATP].

### Sequence alignments

Sequence logos were generated using Geneious. Alignments were generated using the Geneious multiple alignment tool (global alignment with free end gaps, BLOSUM 62 cost matrix).

## Accession numbers

The coordinates of the nucleotide-free BchL structure with the flexible N-terminal region have been deposited in the protein database with PDB ID: 6UYK.

## Data availability

All data are contained within the manuscript. Plasmids used for protein overexpression are available upon request.

## Conflict of interest

The authors declare that they have no conflicts of interest with the contents of this article.
