# Supplementary material for: The flexible N-terminus of BchL autoinhibits activity through interaction with its [4Fe-4S] cluster and released upon ATP binding
Source: J Biol Chem. 2020 Dec 3;296:100107. doi: 10.1074/jbc.RA120.016278 (PMC7948495; doi:10.1074/jbc.RA120.016278)
Supplement: Tables and Figures [file mmc1.docx]

**SUPPLEMENTAL INFORMATION**

**Supplementary Table 1: Crystallographic data processing and refinement statistics.**

| Space Group | I121 |
| --- | --- |
| Unit cell (Å) | a = 92.51 b = 100.92 c = 117.79  α = 90.00° β = 99.27° γ = 90.00° |
| Wavelength (Å) | 1.008 |
| Resolution Range (Å) | 42.20 – 2.60 (2.69-2.60) |
| Total observations | 303261 |
| Unique reflections | 32733 (3125)* |
| R_merge_ | 0.173(0.908) |
| R_pim_ | 0.057(0.413) |
| <I/σ(I)> | 9.2(1.5) |
| CC_1/2_ | 0.990 (0.573) |
| Completeness (%) | 99.5 (95.3) |
| Multiplicity | 9.3 (5.1) |
| R_work_/ R_free_ | 0.231/0.277 |
| Mean B-factor for all atoms (Å^2^) | 85.6 |
| Total number of atoms refined | 7667 |
| Ramachandran favored/allowed (%) | 96.1/3.9 |
| RMSD of bond lengths (Å)/angles (°) | 0.002/0.393 |

* Values in parentheses refer to the highest resolution shell

**Supplemental Table 2. Oligonucleotide primers used in this study**

| 1 | L1 monomer R | 5’ ATTATTCATATGGTCGACTGATTGGAAGTATAGATTCTCTGCGGCCGCTCCATCGAAACCCAGCAACTCGAAAATTTCGCGATCCGGCAGCG 3’ |
| --- | --- | --- |
| 2 | ACYCDuetUp1 | 5’ GGATCTCGACGCTCTCCCT 3’ |
| 3 | L2 monomer F | 5’ AATAATCATATGGAGAATCTATACTTCCAATCAAAGCTTATGAGCCCGAAAGACTTACG ATA CCG ACC GGA GCG GAT GGCGAGGGCTCGGTCCAGGTGC 3’ |

**Supplementary Table 3: Sequence coverage (2-297) of different chains**

| **Chain** | **Coverage (%)** | **N-terminus** | **C-terminus** | **Breaks (C-N)** |
| --- | --- | --- | --- | --- |
| A | 86.1 | 30 | 290 | 210-217 |
| B | 90.5 | 28 | 295 | N/A |
| C | 90.5 | 16 | 293 | 234-240 |
| D | 84.1 | 30 | 292 | 212-217  233-244 |

**Supplemental Table 4. Sources of sequences used for BchL alignment**

**Supplemental Table 5. Sources of sequences used for ChlL alignment**

**Supplemental Methods**

*Construction of L_1_-L_2_ linked-L-protein constructs:* We used a previously described BchL-RSF-Duet 1 plasmid backbone for cloning the linked-BchL construct.^1^ The two consecutive BchL ORFs are designated L_1_ and L_2_, respectively. For construct 1 – ‘BchL_1_-pRSF-Duet1’, the ORF encoding the L_1_ monomer was PCR amplified using primers #1 and #2 (Supplemental Table 2), designed to eliminate the stop codon at the end of the *BchL* ORF and insert a TEV protease recognition sequence. The PCR product was engineered into an empty RSF-Duet 1 plasmid using BamHI and NdeI. For construct 2 – ‘BchL_2_-pRSF-Duet1’, the ORF encoding the L_2_ monomer was similarly PCR amplified using primer #3 (Supplemental Table 2) and a T7 reverse primer and engineered into an empty pRSF Duet1 plasmid using Nde1 and BamHI. This ORF encodes the linker between the L_1_ and L_2_ genes, as well as a second TEV protease recognition sequence. The flanking TEV sites were designed to remove the intervening linker, post-purification with TEV protease, if necessary. For construct 3 – ‘BchL_1_-L_2_-pRSF-Duet1’, the L_2_ ORF was cut from BchL_2_-pRSF-Duet1 with NdeI and BamHI and engineered into BchL_1_-pRSF-Duet1 using the same restriction sites. Lys44 to Ala substitutions were first introduced into the respective BchL_1_-pRSF-Duet1 or BchL_2_-pRSF-Duet1 plasmids using Q5 mutagenesis and subsequently subcloned into the BchL_1_-L_2_-pRSF-Duet1 plasmid as described above. The subsequent iterations of the linked L constructs (Supplemental Fig. S9c), where the linker lengths were varied, were synthesized as codon-optimized synthetic genes (Genscript Inc.).


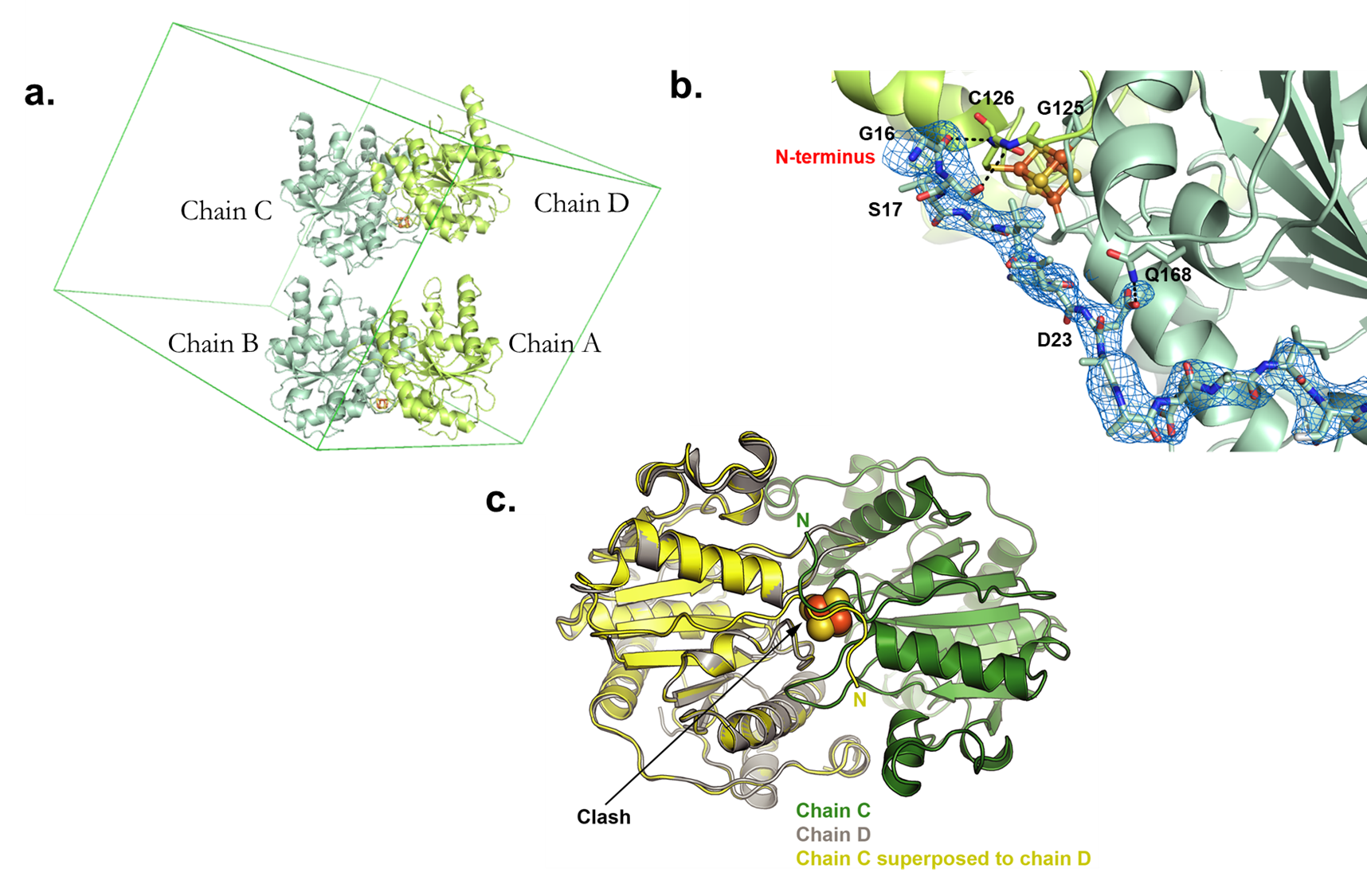
**Supplemental Figure 1. Crystal structure of nucleotide-free BchL.**

**(a)** The asymmetric unit of the crystal structure comprises two dimers of BchL chains: AB and CD. The unit cell is outlined in green. **(b)** 2mFo-DFc electron density for the N-terminus in chain C (shown as blue mesh, contoured at 1.0σ). Continuous density is visible for the tail region up to residue 16. Based on the 2YNM structure, twelve conserved residues are thought to be involved in docking with BchNB (Gly123, Cys126, Tyr129, Gly161, Gly162, Gln168, Asn200, Tyr201). Two of these residues are involved in direct polar contacts with the flexible N-terminal region: Gly16 and Asp23 of the N-terminus are within hydrogen-bonding distances of Cys126 and Gln168, respectively. Additionally, Ser17 is well positioned to hydrogen bond with the backbone amide of Gly125. **(c)** Superposition of chain C to chain D indicates that the N-terminus would clash if present in this conformation in both monomers. Sequence coverage of the four BchL chains in the unit cell is noted in Supplemental Table 3.


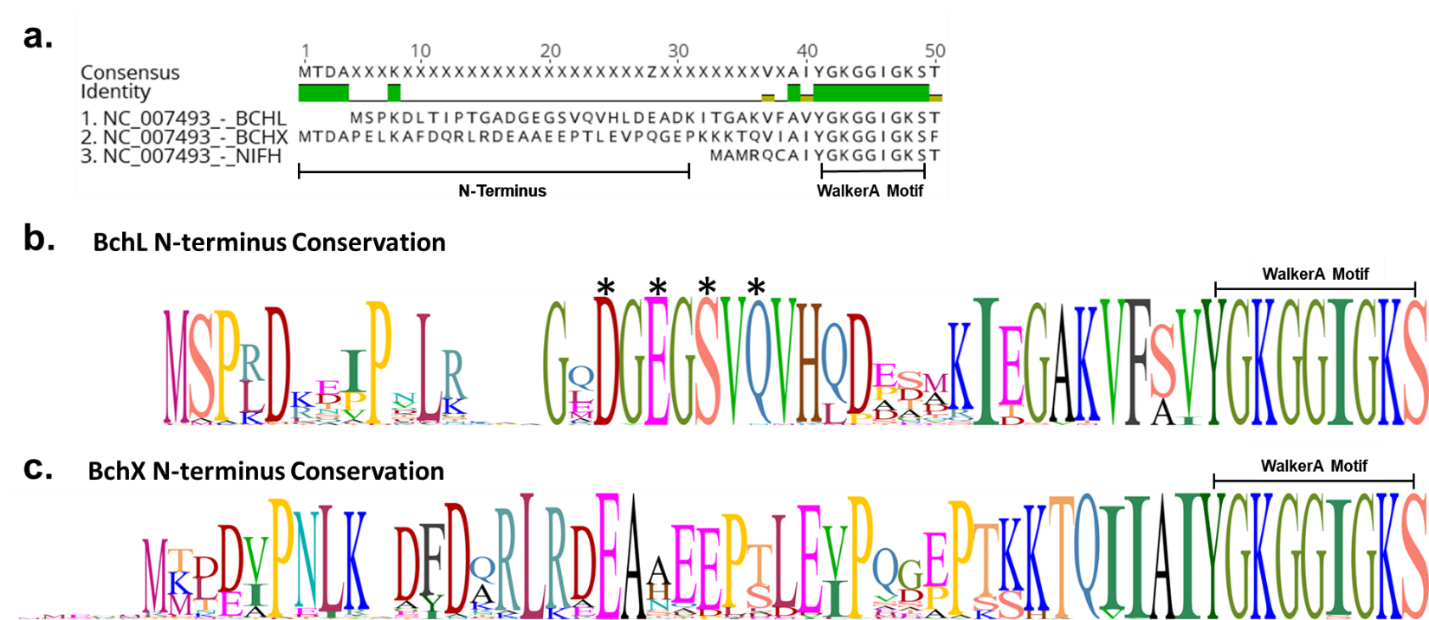


**Supplemental Figure 2. Conservation of the N-terminal flexible region.**

**(a)** Sequence alignment BchL, BchX, and NifH (the nitrogenase Fe-protein). N-terminus length highlighted for BchL and BchX. Walker A motif sequence conservation is also labeled. **(b)** Sequence logo of the N-terminus of BchL generated from alignment of n=89 species. Letter height represents sequence conservation. Critical amino acids for capping and generation of BchL^S17A^ and BchL^4A^ are labeled with asterisks. **(c)** Sequence logo of the N-terminus of BchX generated from alignment of n=89 species. Letter height represents sequence conservation. Figure demonstrates lack of conservation in N-terminus of BchX. NifH N-terminus conservation is not shown as it lacks enough residues to potentially cap its cluster. The list of organisms used for the alignment of BchL are listed in Supplemental Table 3.

**
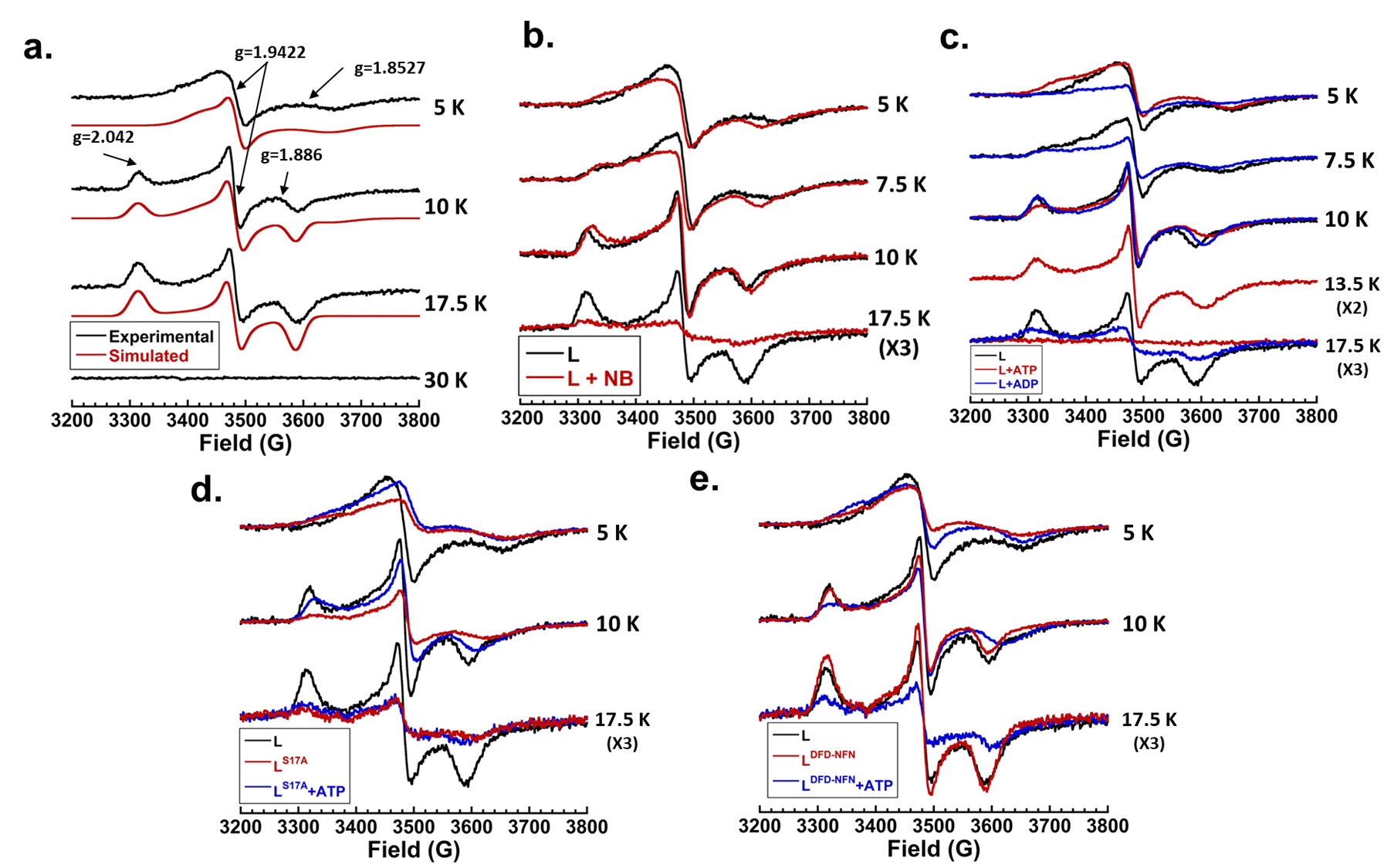
**

**Supplemental Figure 3. EPR analysis of wild-type and variant BchL proteins show ATP-dependent changes in the [4Fe-4S] cluster.**

**(a)** Experimentally determined (black) and simulated (red) EPR spectra of BchL at 5 K, 10 K, 17.5 K, and 30 K. No absorbance was detected at 35K, typical of [4Fe-4S] clusters. There appear to be two species, a fast-relaxing almost-axial species (g = [1.9750 1.9422 1.8527]) and a slower-relaxing rhombic species (g = [2.0420 1.9442 1.8860]). The spectrum at 10 K was simulated using a 40 % axial:60 % rhombic mixture. The g-values for the axial signal are highly unusual in having all three values less than 2.0. In addition, the line shape could not be replicated well, probably due to rapid passage artifacts. The rhombic signal, on the other hand, is "well-behaved".  **(b)** EPR spectra of BchL (black traces) and BchL with 1:1 BchNB (red traces) at indicated temperatures. Upon adding BchNB, there are spectral and relaxation changes. The high-field resonance of the axial signal occurs at a higher g-value (lower field) and features due to the g1 of the rhombic signal persist (distorted by rapid passage) at 5 K. The rhombic signal is very similar in shape to that of the BchL protein alone, but in the presence of BchNB, the signal undergoes significant relaxation broadening at 17.5 K. **(c)** EPR spectra of BchL (black traces), BchL incubated with excess ATP (red traces), and BchL incubated with excess ADP (blue traces) at indicated temperatures. ATP incubation produced the most drastic spectral and relaxation changes, particularly at higher temperatures, broadening relaxation almost entirely. ADP incubations produced similar, though not identical effects as BchNB incubation. **(d)** EPR spectra of BchL (black traces), BchL^S17A^ (red traces), and BchL^S17A^ incubated with excess ATP (blue races) at indicated temperatures. The slow relaxing axial species was distorted for BchL^S17A^ compared to wildtype. The fast relaxing species was significantly broadened compared to wild-type and was unaffected by ATP incubation, though the mixed-species signal and axial species were both significantly diminished compared to wt. **(e)** EPR spectra of BchL (black traces), BchL^DFD-NFN^ (red traces), and BchL^DFD-NFN^ incubated with excess ATP (blue traces). BchL^DFD-NFN^ produced spectra similar to wt BchL with some minor distortions in the axial signal. ATP incubation with BchL^DFD-NFN^ produced spectral changes similar to those produced for the wildtype BchL, though to a lesser extent.

**
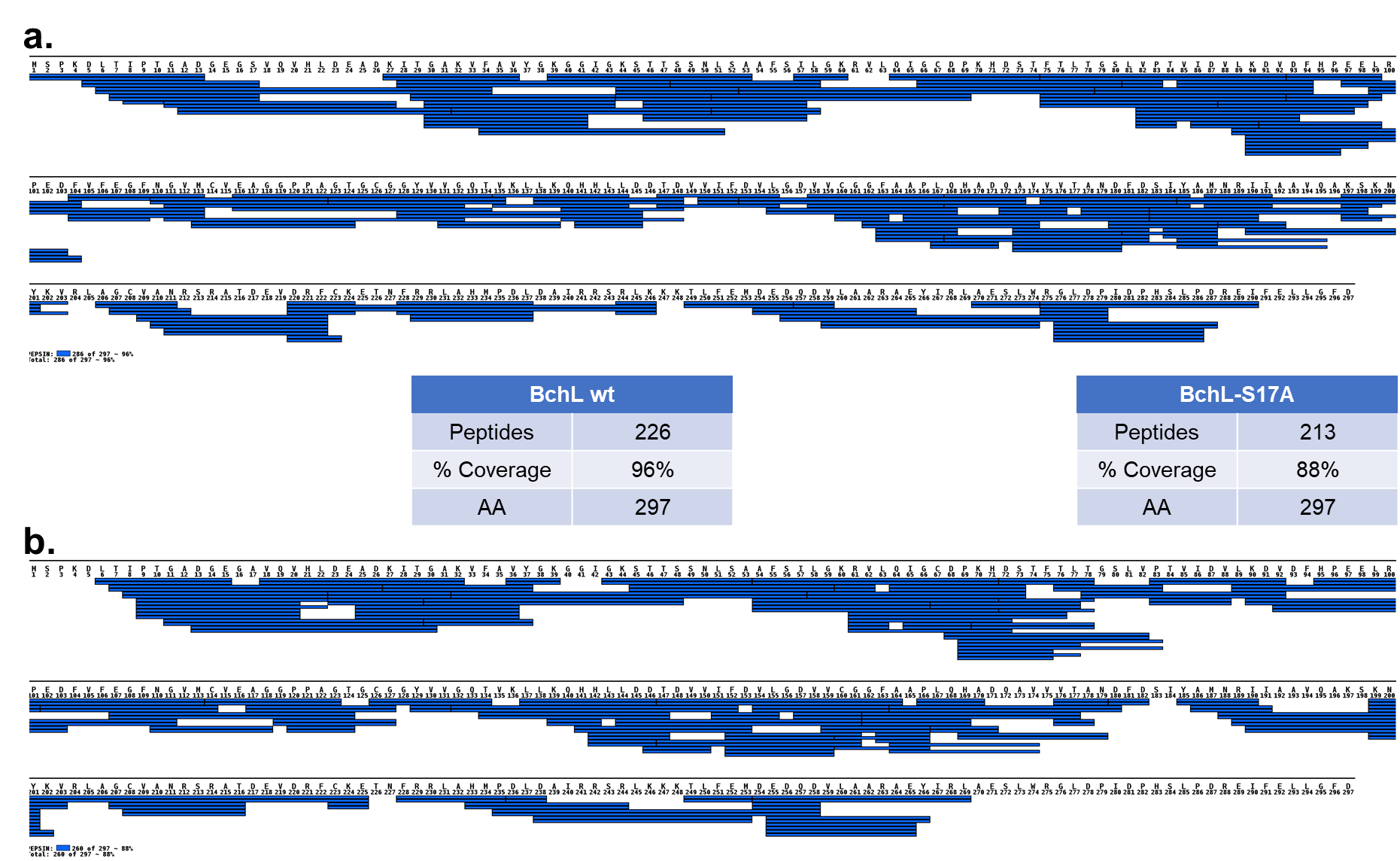
**

**Supplemental Figure 4. Peptide coverage map of BchL and BchL^S17A^.**

Peptides identified in the HDX-MS experiments are presented below the sequence of **(a)** BchL and **(b)** BchL^S17A^. 226 (96 % coverage) and 213 peptides (88 % coverage) were detected for BchL and BchL^S17A^, respectively. Each blue bar represents one peptide an dcorrespond to the respective amino acids denoted in the sequence.

**
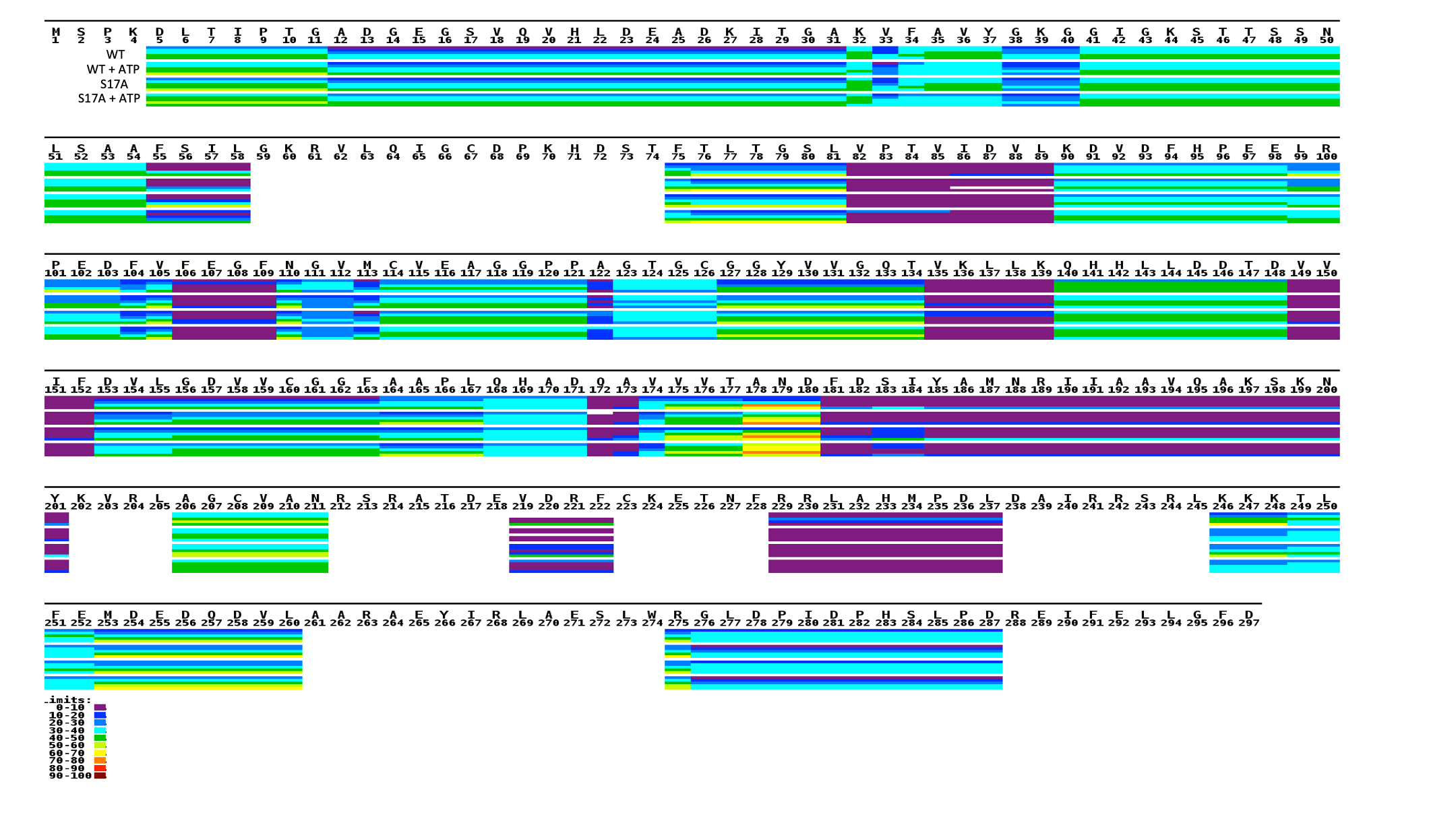
**

**Supplemental Figure 5. ATP-induced conformational changes identified in HDX-MS analysis of BchL and BchL^S17A^.**

Comparison of HDX changes in the peptides from BchL and BchL^S17A^ captured in the absence or presence of ATP. The scale of the changes is color coded as show in the legend. Amino acid sequence of the wild-type is shown above.

**
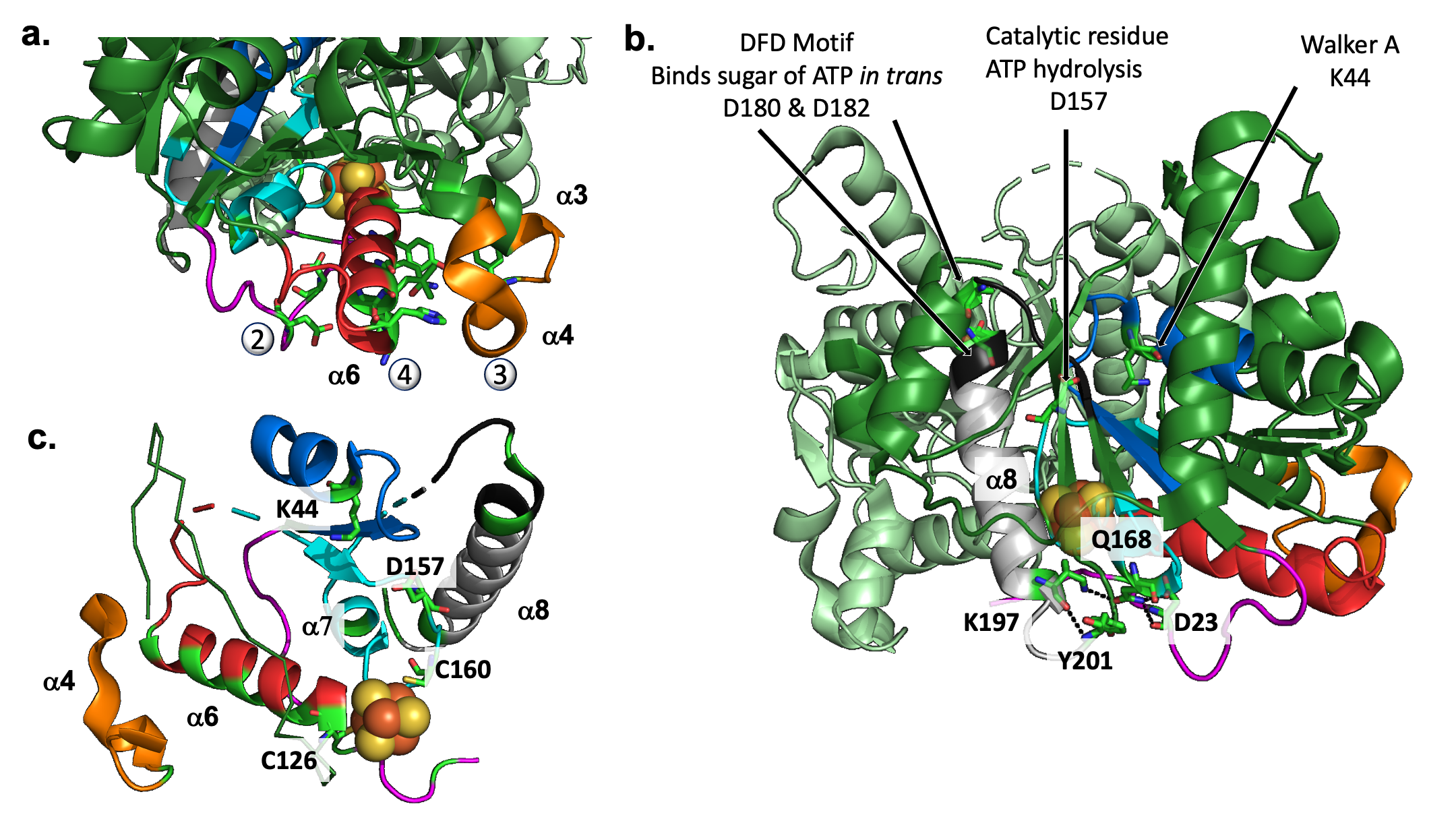
**

**Supplemental Figure 6. Regions of HDX changes in BchL.**

**(a)** Seven regions (numbered 1-7 as denoted) are mapped onto the crystal structure of BchL. Electrostatic and hydrophobic interactions between helices α6 (red), α4 (orange), and the flexible N-terminal region (purple) are shown as sticks. **(b)** The interactions between α6 and the flexible N-terminal region are shown. The long helix α8 connecting the ATP binding region and the region protected by the flexible N-terminal region is shown in black and grey. The grey denotes the lower part of the helix and the black denotes the upper part which houses the DFD motif. **(c)** A cut out of the key helices in BchL are shown. The Walker-A loop is shown in blue, the Walker-B loop is shown in cyan. D157, the catalytic residue for ATP hydrolysis and K44, the residue that coordinates Mg^2+^ binding and the phosphates of ATP is also shown. C126 and C160 covalently coordinate the [4Fe-4S] cluster.


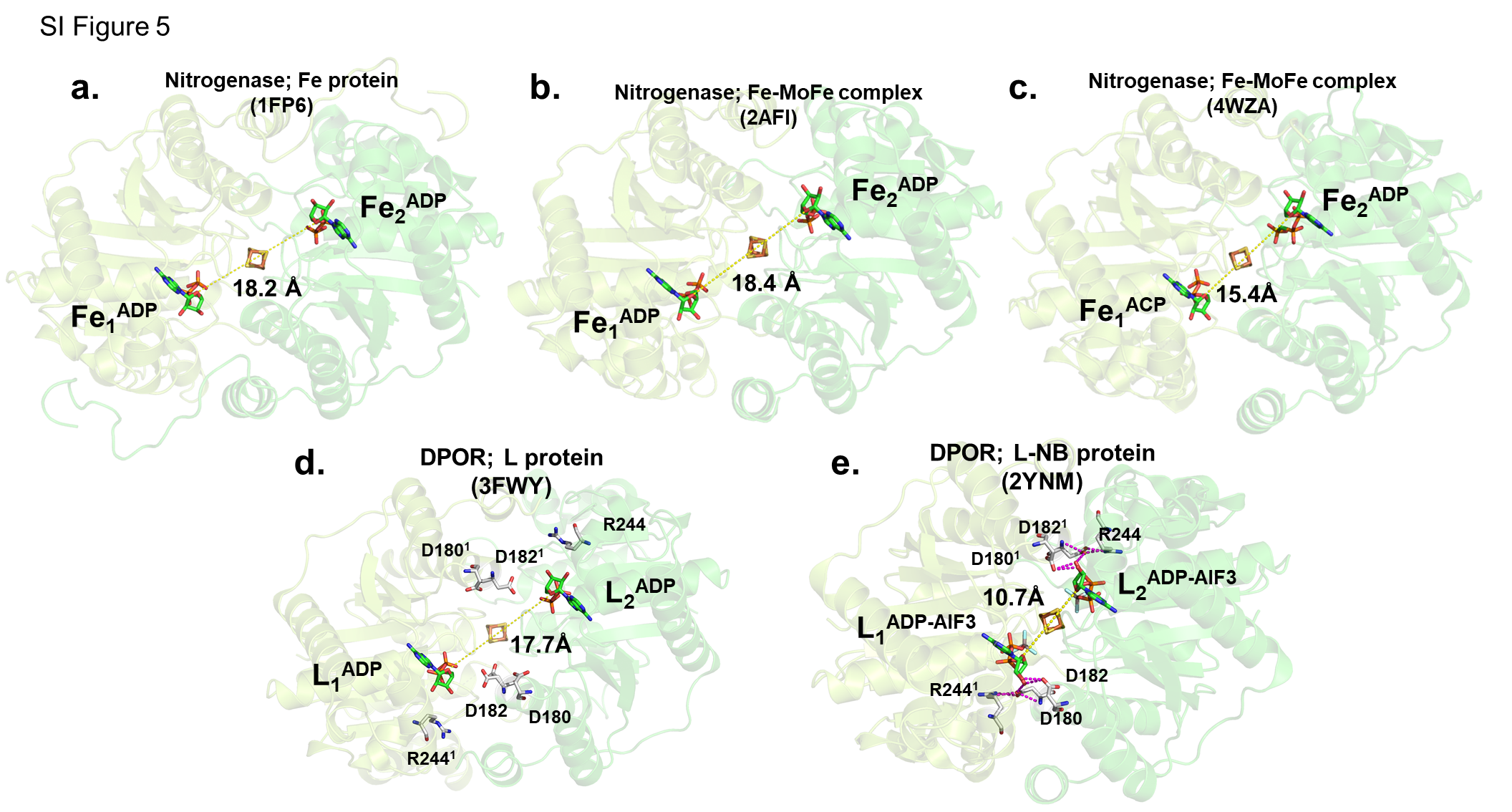


**Supplemental Figure 7**. **Crystal structures of substrate-bound BchL and the Fe-protein from nitrogenase.**

Monomers are colored light and dark green as in previous figures, with highlighted residues and [4Fe-4S] clusters shown as sticks. Distance measurements are shown as dotted yellow lines. **(a)** ADP-bound Fe-protein. **(b)** ADP-bound Fe-protein in complex with MoFe protein. **(c)** ADP- and ACP-bound Fe-protein in complex with MoFe protein. **(d)** ADP-bound BchL. **(e)** ADP-AlF_3_-bound DPOR in complex with BchNB (not shown). BchL in 3FWY is from *R. sphaeroides*. BchL in 2YNM is from *P. marinus*.


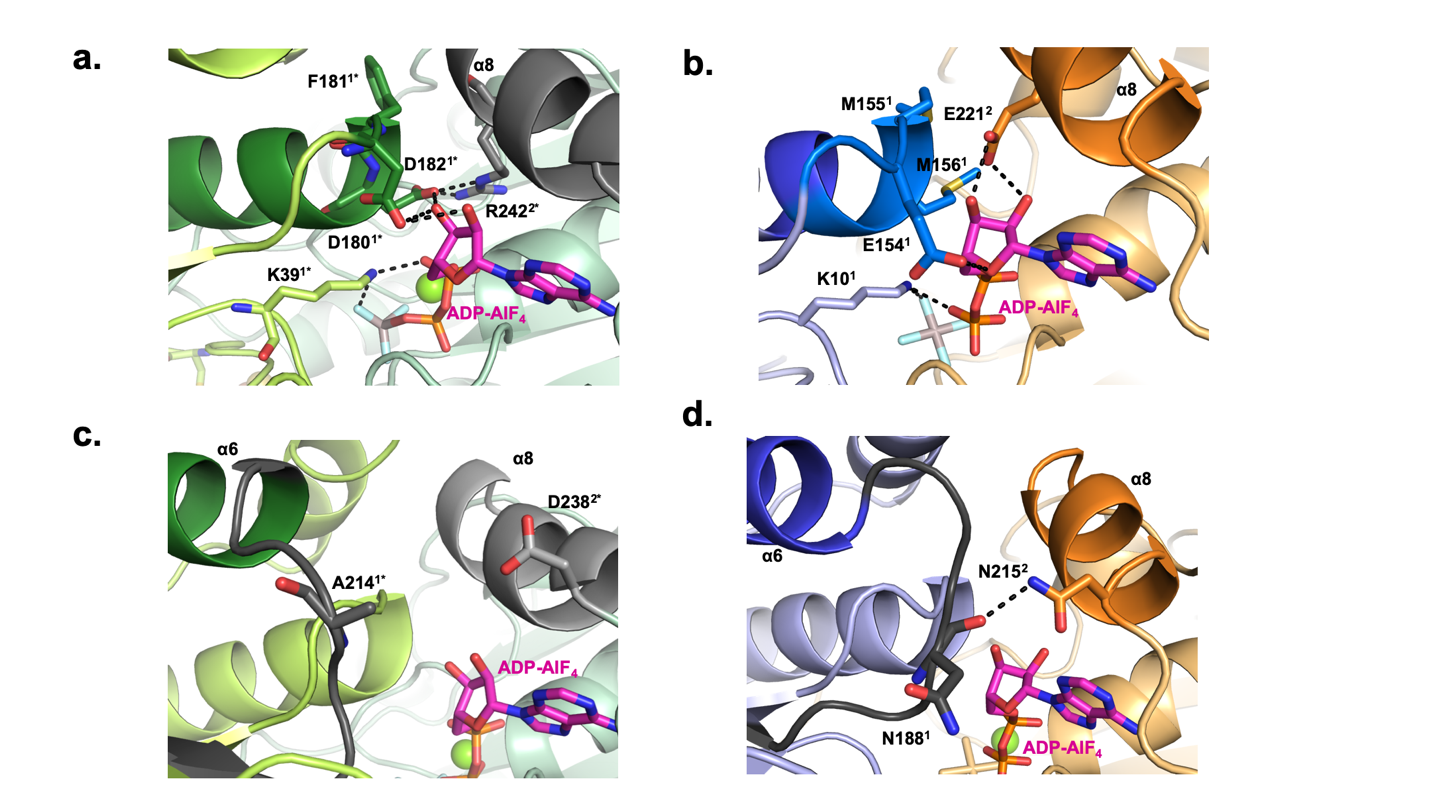


**Supplemental Figure 8. Comparison of nucleotide binding contacts between BchL (DPOR) and Fe-protein (nitrogenase).**

Comparison of inter-subunit interactions observed in structures of the DPOR complex (PDB:2YNM) and nitrogenase (PDB:1N2C). **(a)** The DFD patch of the DPOR L-protein promotes inter-subunit cross stabilization in the ADP-AlF_3_-stabilized complex. **(b)** The corresponding region in *A. vinelandii* nitrogenase consists of Glu154, Met155, and Met156. None of these residues participate in inter-subunit interactions. (c) In DPOR, the loop connecting the α6 helix and β7 strand (dark grey) does not form any interactions with the opposing subunit (light gray). (d). In the nitrogenase complex, the corresponding loop forms a hydrogen-bonding interaction with the side chain of residue Glu215 on the α8 helix of the opposing subunit (orange).


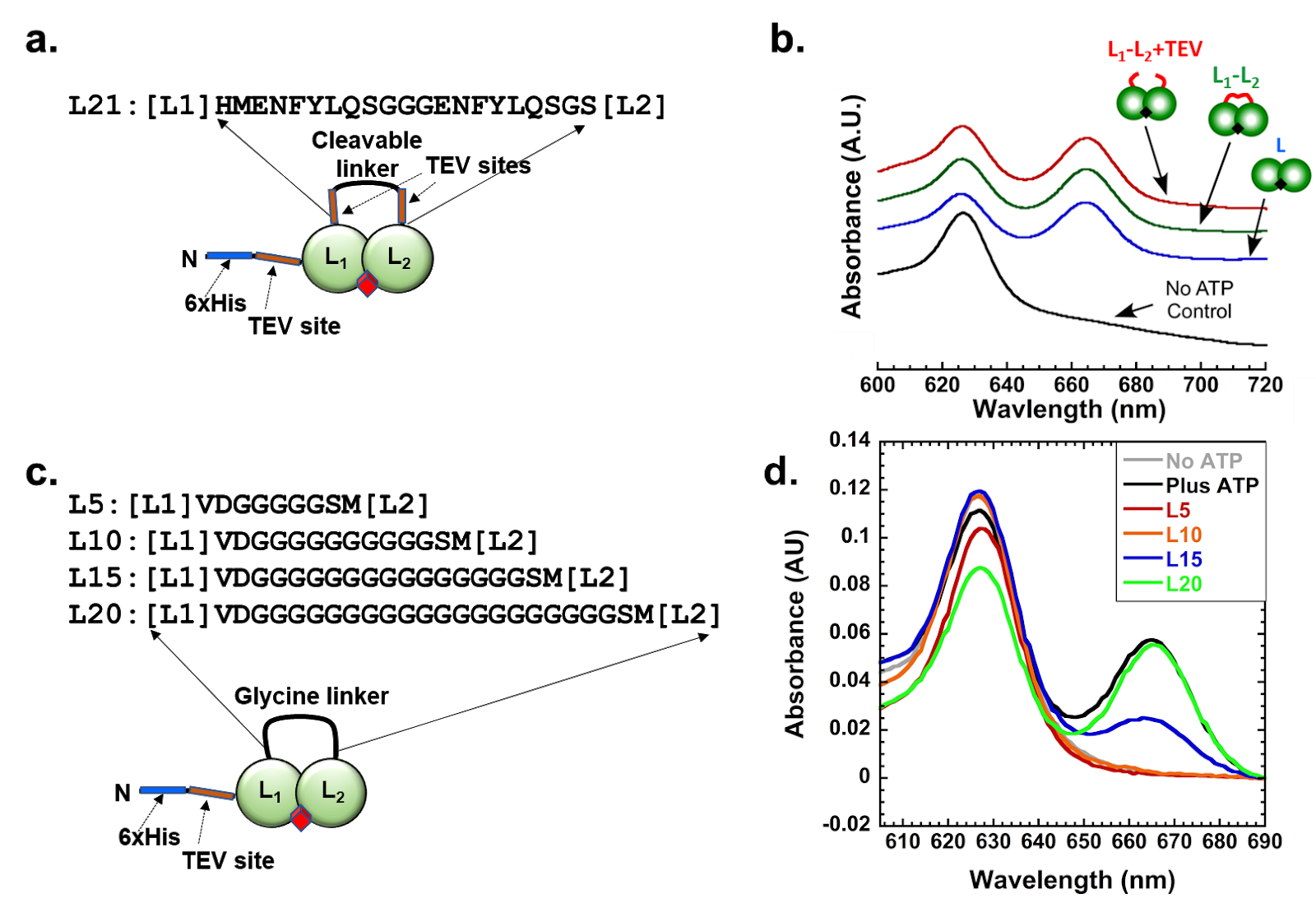


**Supplemental Figure 9: Substrate reduction activity of linked-BchL proteins.**

**(a)** Cartoon representation of the linked BchL construct, and the amino acid sequence of the linker. **(b)** Absorbance plot of acetone extracted Pchlide and Chlide after *in vitro* reaction. Cartoon representations of constructs are shown, and absorbance traces are shifted for clarity. The no-ATP (negative control -black), WT reaction (positive control -blue), linked WT (green), and linked WT after cleavage with TEV protease (red) are shown.  **(c)** Cartoon representation of poly-glycine linkers lacking protease sites, with amino acid sequences of various linker lengths. **(d)** Absorbance plot of acetone extraction of pigments after *in vitro* reaction. The no-ATP negative control and WT reaction positive control shown as grey and black traces respectively. L5, L10, L15, and L20 construct traces are shown as red, orange, blue, and green lines, respectively.


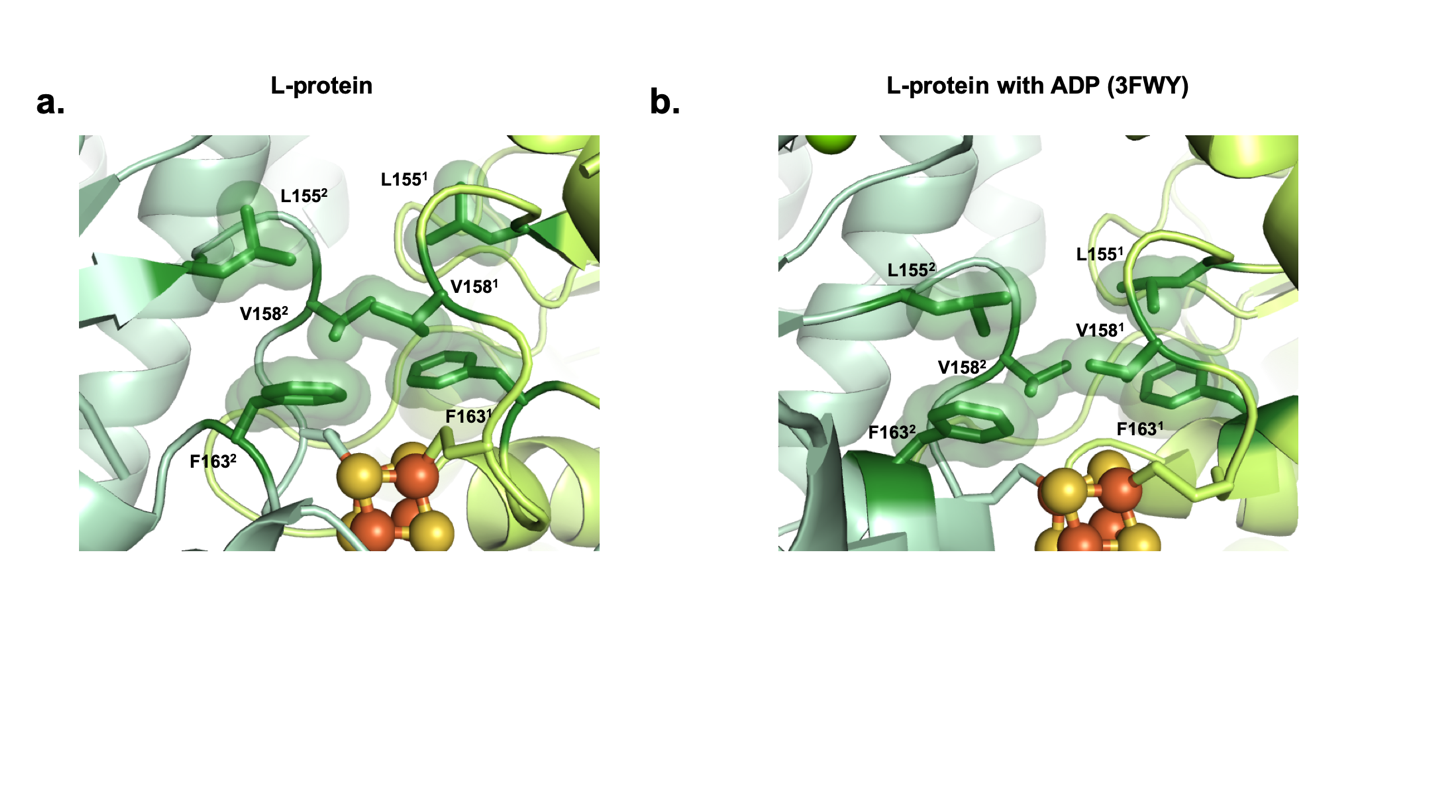


**Supplemental Figure 10:** **Perturbations in the Switch-II region change the local environment around the [4Fe-4S] cluster.**

Highlighted in dark green are the interactions of six hydrophobic residues, Leu155, Val158 and Phe163 on each chain, **(a)** in the absence of nucleotide and **(b)** in the presence of ADP. The environment directly above the [4Fe-4S] cluster becomes less hydrophobic in the presence of ADP, largely as a result of repositioning of the adjacent Phe163 residues relative to the cluster. More hydrophobic environments generally correlate with increased reduction potential for redox-active metal centers, suggesting that the binding of Mg-ADP acts as a form of redox control in BchL by increasing the tendency of the cluster to become oxidized.

**
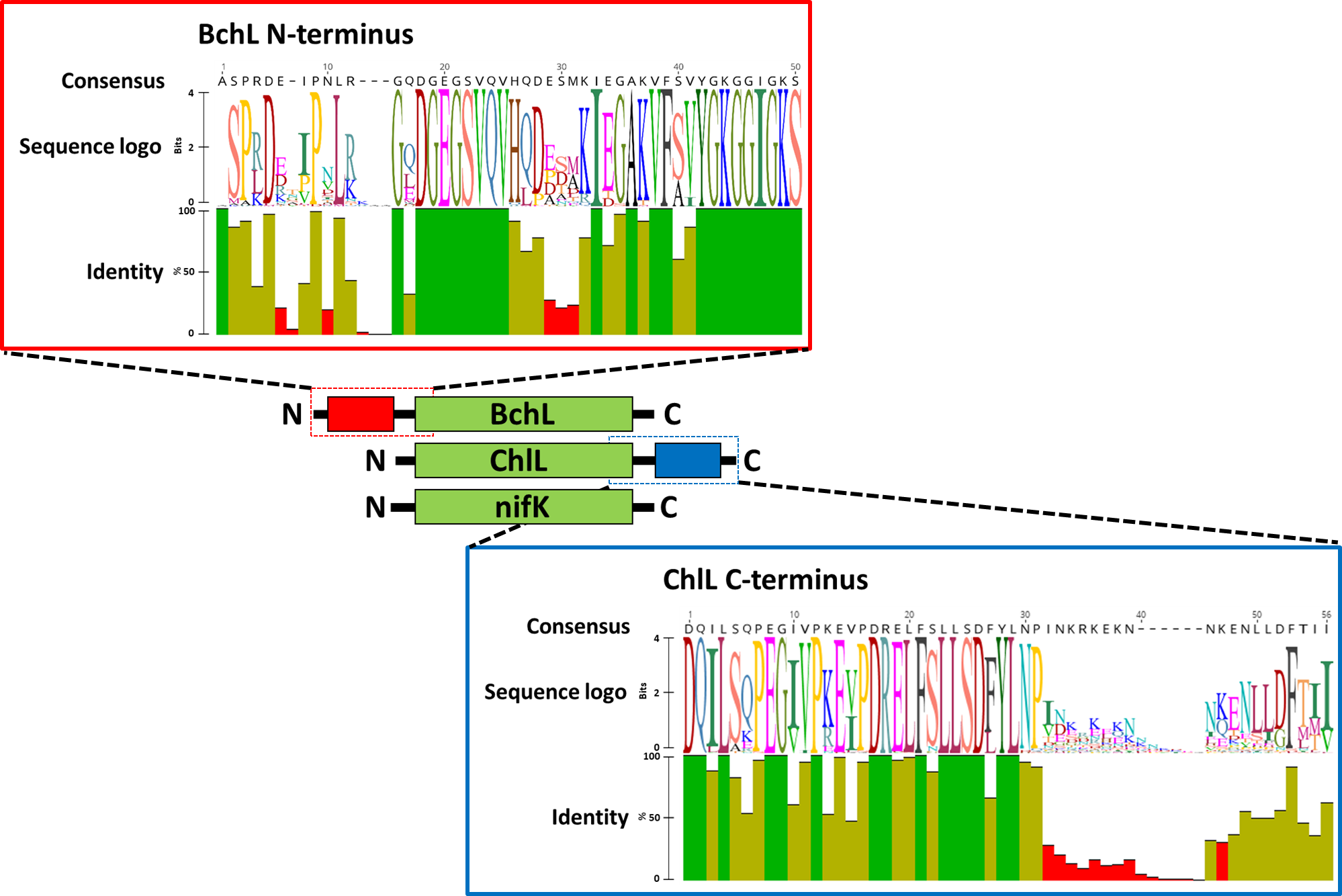
**

**Supplemental Figure 11.**

Sequence conservation and identity within the disordered regions of BchL and ChlL. 97 BchL and 100 ChlL sequences from various organisms were aligned using Geneious alignment tool. Sequence logos from the alignment and the corresponding percent identity within the flexible N-terminal region of BchL and the C-terminal extension of ChlL are depicted. The list of organisms used for the alignment of BchL are listed in Supplemental Table 3. The list of organisms used for the alignment of ChlL are listed in Supplemental Table 4.

**Supplemental References**

1. Corless, E.I., Mettert, E.L., Kiley, P.J. & Antony, E. Elevated Expression of a Functional Suf Pathway in *E. coli* BL21(DE3) Enhances Recombinant Production of an Iron-Sulfur Cluster Protein. *Journal of Bacteriology*, JB.00496-19 (2019).
